# Creation of Cationic Polymeric Nanotrap Featuring High Anion Density and Exceptional Alkaline Stability for Highly Efficient Pertechnetate Removal from Nuclear Waste Streams

**DOI:** 10.1021/acscentsci.3c01323

**Published:** 2024-01-31

**Authors:** Bin Wang, Jie Li, Hongliang Huang, Bin Liang, Yin Zhang, Long Chen, Kui Tan, Zhifang Chai, Shuao Wang, Joshua T. Wright, Robert W. Meulenberg, Shengqian Ma

**Affiliations:** † Department of Chemistry, University of North Texas 1508W Mulberry St, Denton, Texas 76201, United States; ‡ State Key Laboratory of Radiation Medicine and Protection, School for Radiological and Interdisciplinary Sciences (RAD-X), and Collaborative Innovation Center of Radiation Medicine of Jiangsu Higher Education Institutions, Soochow University, Suzhou 215123, China; § State key laboratory of Separation Membranes and Membrane Processes, Tiangong University, Tianjin 300387, China; ∥ Department of Physics, Illinois Institute of Technology, Chicago, Illinois 60616, United States; ⊥ Department of Physics and Astronomy and Frontier Institute for Research in Sensor Technologies, University of Maine, Orono, Maine 04469, United States

## Abstract

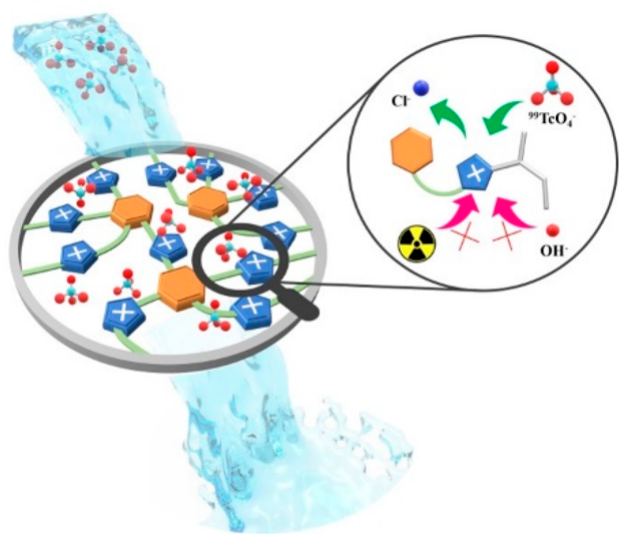

There is an urgent need for highly efficient sorbents capable of selectively removing ^99^TcO_4_^–^ from concentrated alkaline nuclear wastes, which has long been a significant challenge. In this study, we present the design and synthesis of a high-performance adsorbent, CPN-3 (CPN denotes cationic polymeric nanotrap), which achieves excellent ^99^TcO_4_^–^ capture under strong alkaline conditions by incorporating branched alkyl chains on the N3 position of imidazolium units and optimizing the framework anion density within the pores of a cationic polymeric nanotrap. CPN-3 features exceptional stability in harsh alkaline and radioactive environments as well as exhibits fast kinetics, high adsorption capacity, and outstanding selectivity with full reusability and great potential for the cost-effective removal of ^99^TcO_4_^–^/ReO_4_^–^ from contaminated water. Notably, CPN-3 marks a record-high adsorption capacity of 1052 mg/g for ReO_4_^–^ after treatment with 1 M NaOH aqueous solutions for 24 h and demonstrates a rapid removal rate for ^99^TcO_4_^–^ from simulated Hanford and Savannah River Site waste streams. The mechanisms for the superior alkaline stability and ^99^TcO_4_^–^ capture performances of CPN-3 are investigated through combined experimental and computational studies. This work suggests an alternative perspective for designing functional materials to address nuclear waste management.

## Abstract 2

An alkaline-stable cationic polymeric nanotrap was developed for efficient removal of ^99^TcO_4_^−^ from nuclear wastewater.

## Introduction

Nuclear energy, a sustainable and ecofriendly power source, has a low fuel utilization rate of only 0.6%.^1^ To improve this, countries are actively exploring reprocessing spent nuclear fuel as a promising solution.^2^ However, the conventional method, plutonium uranium reduction extraction (PUREX), faces challenges like high costs and equipment corrosion.^3^ Urgent investigation of cost-effective and environmentally benign alternatives, such as carbonate extraction (CARBEX), is crucial. CARBEX effectively separates fission products, providing a viable solution for managing alkaline high-level radioactive waste without PUREX’s limitations.^4^ On the other hand, there is much historically accumulated alkaline high-level radioactive waste (HLW) that needs urgent treatment, such as the 18 000 m^3^ stored in the Mayak Production Association and millions of gallons stored in the Hanford Site in Washington State and the Savannah River Site (SRS) in South Carolina.^5−7^ An essential challenge lies in the efficient separation of fission products under highly alkaline conditions. Notably, technetium-99 (^99^Tc) emerges as a significant fission product abundantly present in alkaline waste. In aqueous solutions, ^99^Tc is primarily found as the pertechnetate anion (^99^TcO_4_^–^), which has high water solubility, toxicity, and environmental mobility, leading to its depth removal by the traditional precipitation method difficult.^8,9^ Consequently, a primary research focus is the development of a selective removal technique targeting ^99^TcO_4_^–^ in alkaline waste.

Ion exchange is considered one of the most promising technologies for selectively capturing and separating ^99^TcO_4_^–^ due to its high efficiency and ease of operation. Various ion-exchange materials have been tested for ^99^TcO_4_^–^ removal.^8^ Cationic inorganic materials, such as layered double hydroxides (LDHs),^10^ framework hydroxide,^11^ and thorium borate,^12,13^ are advantageous in terms of easy preparation and low cost, but their low capture capacity and poor selectivity toward ^99^TcO_4_^–^ hinder their practical application in alkaline nuclear waste streams. Commercially available organic anion-exchange resins such as Puroilte A530E and A532E exhibit good sorption selectivity toward ^99^TcO_4_^–^; however, slow sorption kinetics and poor radiation resistance are inevitable issues.^14^ Cationic organic–inorganic hybrid materials, such as metal–organic frameworks (MOFs), exhibit a high adsorption capacity, fast sorption kinetics, and high selectivity toward ^99^TcO_4_^–^. However, the coordination bonds used for the construction of MOFs are usually vulnerable, which can result in the collapse of the framework under high-alkaline and salinity conditions.^15−19^ Additionally, the high cost of MOFs presents a barrier to their practical application. Therefore, there is a crucial need for the development of highly efficient, alkaline-stable, and cost-effective sorbents for ^99^TcO_4_^–^ removal from alkaline nuclear wastes. Another factor that needs to be noted is that the concentration of anions in the adsorbent directly affects the sorption performance for ^99^TcO_4_^–^ when the ion-exchange method was used. To increase the anion capacity in framework materials, modifications can be made by reducing the size of noncharged fragments in the repeating units while preserving the charged fragments and increasing the number of charged fragments in the repeating units.

**Figure 1:**
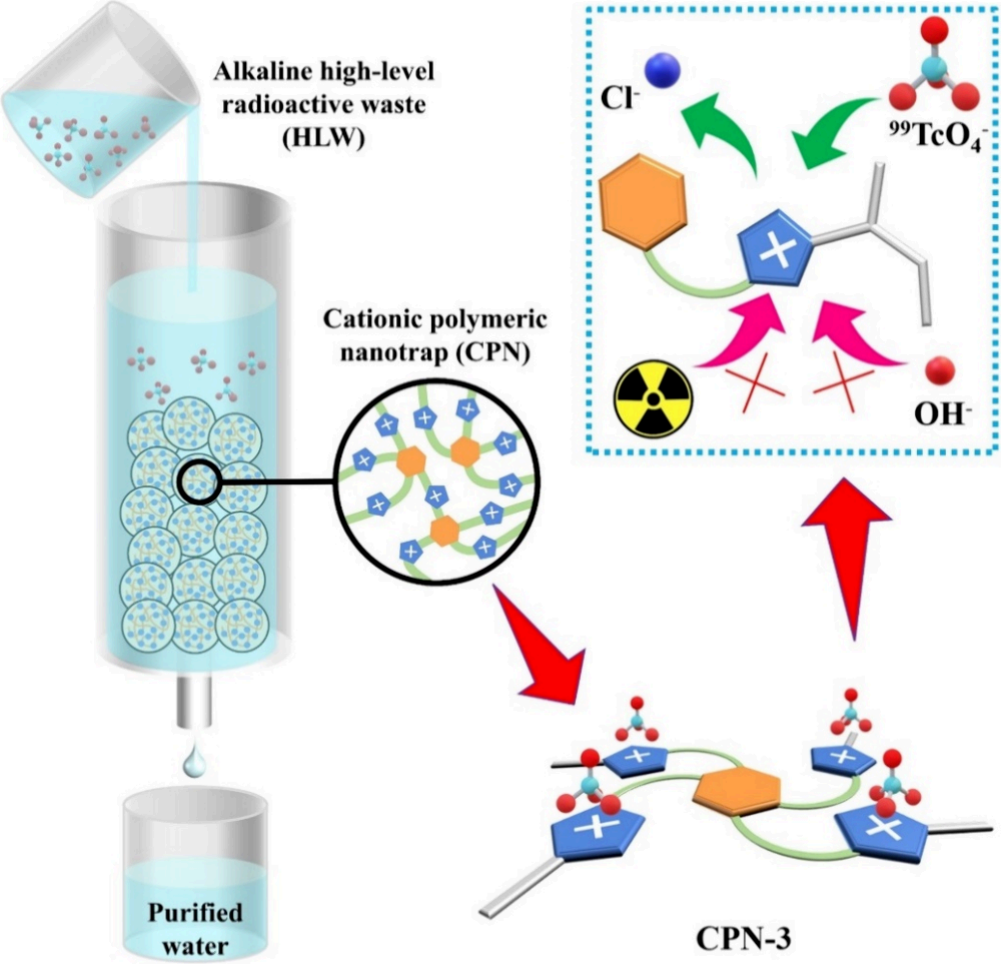
Proposed strategy for the construction of high-alkaline resistant imidazolium-based CPNs for the capture of ^99^TcO_4_^–^ from high-alkaline HLW.

Cationic polymeric nanotraps (CPNs), a subclass of porous organic polymers (POPs), are constructed by utilizing cationic organic building blocks through covalent bonds or postsynthesis functionalization of a neutral POP material with cationic groups. With their cationic framework, high surface area, and customizable structures and functions, CPNs are highly regarded as an excellent platform for efficiently adsorbing ^99^TcO_4_^–^/ReO_4_^–^ (the nonradiative surrogate of ^99^TcO_4_^–^) from wastewater.^20−35^ The CPNs constructed from imidazolium-based organic building units show excellent selective adsorption performance toward ^99^TcO_4_^–^/ReO_4_^–^.^26,28,35,36^ However, imidazolium moieties are inherently unstable in highly alkaline conditions. In alkaline environments, OH^–^ as a nucleophilic reagent preferentially attacks the C2 position of imidazolium ions, leading to the degradation of the imidazolium ring.^37−41^ This drawback directly affects the adsorption of ^99^TcO_4_^–^/ReO_4_^–^ by imidazolium-based CPNs under alkaline conditions. For example, two such adsorbents reported by us, SCU-CPN-1 and SCU-CPN-2, exhibited a significant decrease in adsorption capacity for ReO_4_^–^ after immersion in a NaOH solution, indicating their structural instability in highly alkaline environments.^42^ The introduction of hydrophobic or bulky substituents at positions N1, C2, N3, C4, and C5 of the imidazolium ring can effectively enhance its stability in alkaline solutions.^37−40^ We incorporated this strategy into the construction of imidazolium-based adsorbents and successfully obtained an adsorbent named SCU-CPN-4 with excellent alkaline stability for ^99^TcO_4_^–^/ReO_4_^–^. However, while this modification increases material stability, it significantly reduces the concentration of the anion in the material, resulting in a low ReO_4_^–^ adsorption capacity of 437 mg/g.^42^ It is reported that introducing branched alkyl groups (e.g., isopropyl) solely at the N3 position of the imidazolium ion can greatly enhance the alkaline stability of the resulting materials.^39^ Moreover, N3-substituted imidazolium cations can be synthesized in a one-step process through simple nucleophilic substitution reactions, offering better economic feasibility. Bearing this in mind, we present a strategy for constructing highly alkaline-stable imidazolium-based adsorbents for efficient capture of ^99^TcO_4_^–^/ReO_4_^–^ under strong alkaline conditions (Figure 1).

This strategy involves introducing branched alkyl chains on the N3 of imidazolium units and increasing the density of counteranions within the pores of CPNs. The introduction of alkyl chains protects the imidazolium units from attack by OH^–^ groups, while the high anion density ensures a high adsorption capacity for ^99^TcO_4_^–^/ReO_4_^–^. Furthermore, the flexible and hierarchical pore structure of CPNs facilitates mass transfer, promoting sorption kinetics. As a proof of concept, we report in this contribution the preparation of a series of monomers with controlled numbers of imidazolium units based on the quaternization reaction between the 1-vinyl-1H-imidazole and benzene ring with different benzyl bromide substituents. Through a subsequent free-radical polymerization reaction and ion exchange with a NaCl solution, a series of CPNs with controllable anion densities, named CPN-1, -2, and -3, were obtained. The theoretical anion density increases as the number of imidazolium units increases. Additionally, the aliphatic carbon chains on N3 of the imidazolium unit, generated through free-radical polymerization, play a protective role in shielding the imidazolium from attacks by OH^–^ groups. CPN-3, with the highest anion density, exhibits the highest maximum adsorption capacity of 1282 mg/g toward ReO_4_^–^ among the three materials. Also, CPN-3 has excellent chemical stability, and its adsorption toward ReO_4_^–^ can be well-retained after treatment with 1 M NaOH aqueous solutions and 200 kGy γ-ray radioactivity. It showed an all-time ReO_4_^–^ adsorption record of 1050 mg/g after being treated with 1 M NaOH solutions for 24 h. Moreover, CPN-3 demonstrates excellent performance in the removal of ^99^TcO_4_^–^ from simulated Hanford and SRS waste streams and has low material cost compared to the benchmark ^99^TcO_4_^–^ sorbents, which makes it attractive for direct removal of ^99^TcO_4_^–^ from legacy nuclear waste streams. Examination studies and density functional theory (DFT) calculations undisputedly reveal the mechanisms behind the remarkable alkaline stability of CPN-3 and its superior separation capability toward ^99^TcO_4_^–^.

## Results and Discussion

### Design, Synthesis, and Characterizations

**Figure 2:**
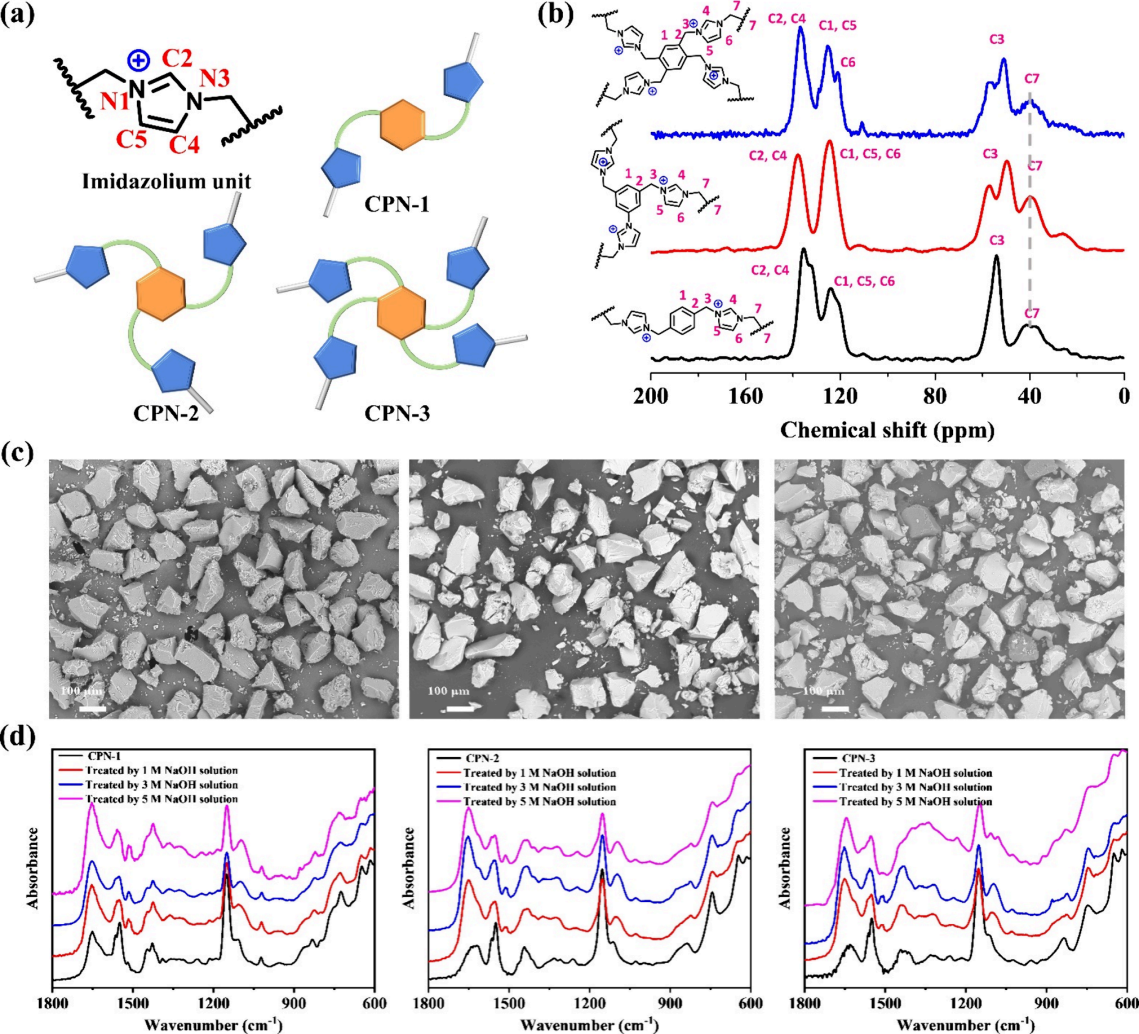
(a) Imidazolium unit and the repeating units in CPN-1, CPN-2, and CPN-3; (b) ^13^C CP-MAS solid-state NMR spectra of CPN-1, CPN-2, and CPN-3; (c) SEM images of CPN-1 (left), CPN-2 (middle), and CPN-3 (right); and (d) FT-IR spectra of CPN-1, CPN-2, and CPN-3 before and after being treated with NaOH aqueous solutions.

Ion exchange is a widely recognized mechanism for the efficient adsorptive removal of ^99^TcO_4_^–^/ReO_4_^–^ from water, where the density of anions present within the material’s framework plays a significant role in enhancing the adsorption capacity of ^99^TcO_4_^–^/ReO_4_^–^. Consequently, it is unsurprising to observe a robust positive correlation between the adsorption capacity and the density of anions (Figure S5 and Table S1). Nonetheless, this correlation has long been disregarded in the development of high-performance ^99^TcO_4_^–^/ReO_4_^–^ sorbents. On the other hand, the introduction of branched alkyl groups at the N3 position is an effective strategy for preventing the degradation of imidazolium units under alkaline conditions. Taking these considerations into account, our initial approach involves regulating the anion density of the monomers through a quaternization reaction between 1-vinyl-1H-imidazole and a benzene ring with different benzyl bromide substituents and named M-1, M-2, and M-3, respectively (Figures S1–3). The successful synthesis of these monomers was confirmed through proton nuclear magnetic resonance (^1^H NMR) spectroscopy (Figure S1–3) analyses. Subsequently, the monomers were polymerized in the presence of azobis(isobutyronitrile) (AIBN) in dimethylformamide (DMF) aqueous solution at 100 °C. Following ion exchange with Cl^–^ in NaCl aqueous solutions, the polymers were obtained in quantitative yield, denoted as CPN-1, CPN-2, and CPN-3, respectively (Figures 2a, S4).^43,44^ Theoretical Cl^–^ densities in CPN-1, CPN-2, and CPN-3 were calculated to be 5.50, 5.93, and 6.17 mmol/g, respectively. These values are comparable to or higher than those of other benchmark ^99^TcO_4_^–^ sorbents, suggesting the potential applications of these materials in the adsorptive removal of ^99^TcO_4_^–^ through ion-exchange processes (Figure S5, Table S1). The Fourier transform infrared (FT-IR) spectra of CPN-1, CPN-2, and CPN-3, along with their corresponding monomers, are presented in Figures S7–9. In the monomers, the identification of a carbon–carbon double bond through stretching mode ν(C=C) is obscured by the occurrence of phenyl and azole ring stretching vibrations in the same region. However, the presence of the alkene group is best characterized by the CH deformation modes (δ), which are manifested as strong absorption features in the range of 900–950 cm^–1^.^45^ The absence of these δ(=CH, =CH_2_) bands upon formation of the polymers indicates the completion of the polymerization reaction of the monomers (Figures S7–9). Figure 2b shows the solid-state ^13^C cross-polarization with magic-angle spinning (CP-MAS) NMR spectra, which identifies a new broad resonance at around 40.0 ppm for all three CPNs, confirming the presence of alkyl carbon atoms and further indicating the successful construction of the polymer. The complete anion exchange, substituting Br^–^ with Cl^–^, was confirmed through energy dispersive X-ray spectroscopy (EDS) analysis, as evidenced by the invisible Br signals in the EDS spectrum (Figures S10–12). Scanning electron microscopy (SEM) revealed similar morphologies for all three CPNs (Figure 2c).

### Alkaline Stability

We then conducted a systematic investigation into the alkaline stability of the three CPNs while also assessing the alkaline stability of their corresponding monomers for comparative purposes. Since the monomers are soluble in water, ^1^H NMR was used to characterize their alkaline stability. We noticed a significant decrease in the intensity of the H atoms associated with the C4 and C5 positions of the imidazolium unit in the M-1 monomer after only 5 min of exposure to 1 M NaOH D_2_O solutions. After 60 min, these H atoms completely vanished, while a new peak emerged at around 7.3 ppm. Additionally, 3 days later, we observed the formation of insoluble solid material in the NMR tube (Figure S14). These findings suggest that M-1 undergoes degradation under high-alkaline conditions, which aligns with what has been previously reported in the literature.^37−40^ With an increase in the number of imidazolium units, we observed an accelerated rate of degradation. Specifically, in the case of M-3, degradation was particularly pronounced. After just 5 min of contact with 1 M NaOH D_2_O solutions, the signals corresponding to the H atoms of M-3 completely disappeared, indicating its remarkably diminished stability under alkaline conditions (Figure S16). With the help of attenuated total reflection (ATR) technology, we were able to obtain the IR spectra of the three monomers before and after treatment with NaOH aqueous solution. Figure S17 shows that after soaking in 1 M NaOH aqueous solution for 60 min, there is a significant decrease in the intensity of the ν(CH)_Ph_ and δ(CH) peaks for M-1. These peaks completely disappear when the NaOH aqueous solution concentration reaches 5 M. The other two monomers also exhibit similar changes in their spectra. These observations further confirm the instability of the three monomers in NaOH aqueous solution, which is consistent with the analysis of the ^1^H NMR data. The alkaline stability of the CPNs was investigated using FT-IR spectroscopy due to their insolubility in water. Figure 2d demonstrates that even after exposure to 5 M NaOH aqueous solutions for 24 h, the FT-IR spectra of CPN-1, CPN-2, and CPN-3 remained almost identical compared to the pristine samples, indicating their exceptional stability when subjected to strong alkaline conditions. Furthermore, SEM images of the three samples after being immersed in 1, 3, and 5 M NaOH aqueous solutions show that their morphology remains unchanged, with no noticeable erosion marks observed on the surface (Figure S18). This further confirms their exceptional stability in alkaline solutions. The remarkable stability of the three CPNs can be attributed to the following reasons. First, the polymeric network of the CPNs is insoluble in water, which limits the contact between the imidazolium units and the OH^–^ ions compared to the soluble monomers. Second, the alkyl chains formed through the free-radical polymerization of olefinic bonds play a crucial role in further safeguarding the imidazolium units from attack by OH^–^ ions by providing local hydrophobic environments. The combination of these two factors synergistically contributes to the exceptional stability of the three materials. The cationic framework with high anion density, combined with the remarkable resistance to alkaline conditions exhibited by the CPNs, encourages further investigation into their potential for effectively removing ^99^TcO_4_^–^ from HLW streams.

### Sorption Isotherm Analysis

**Figure 3:**
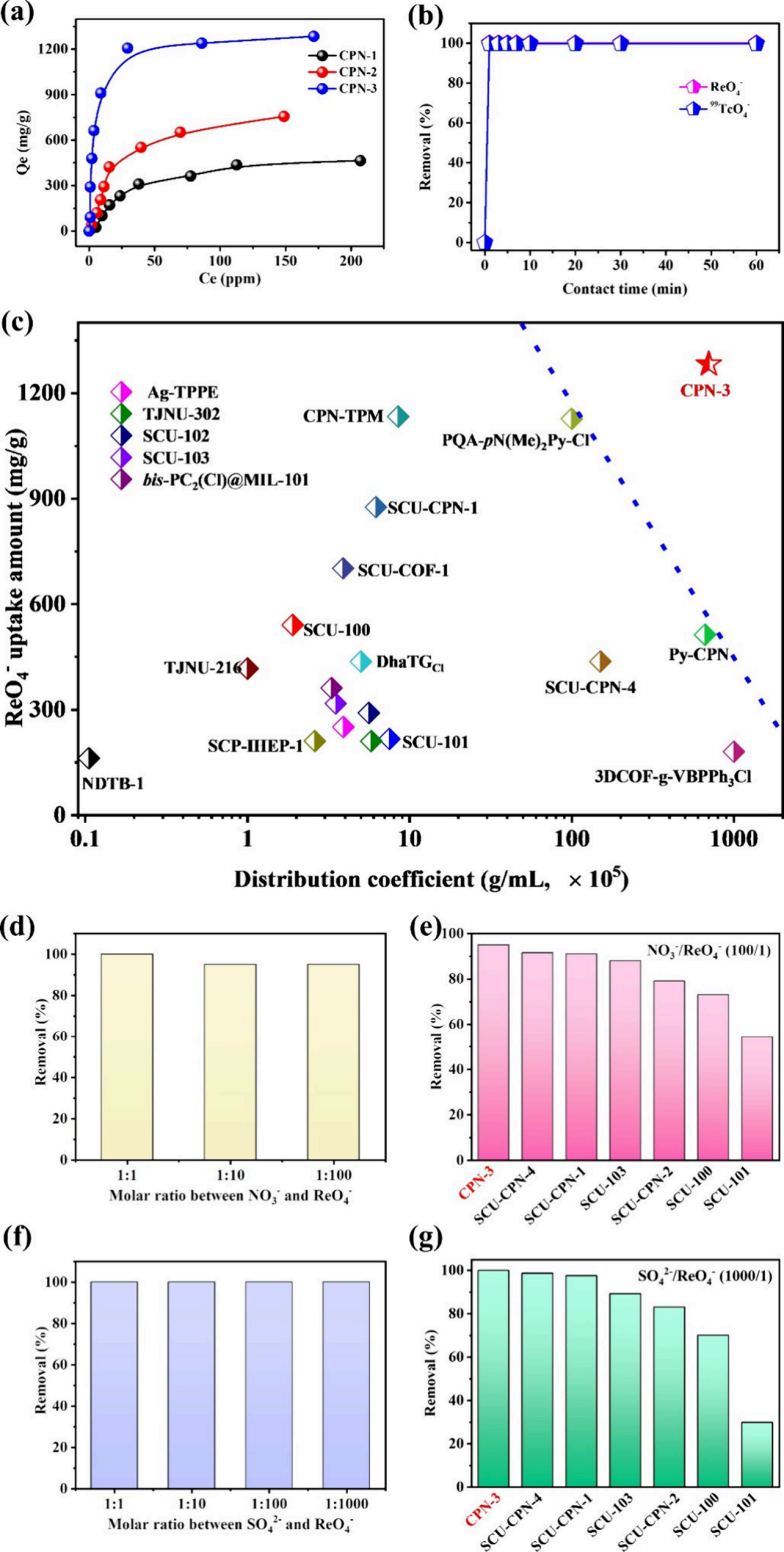
(a) Sorption isotherm of the CPNs for ReO_4_^–^ uptake. Condition: *M*_sorbent_/*V*_solution_ = 0.2 g/L and contact time = 12 h. (b) Sorption kinetics of ^99^TcO_4_^–^/ReO_4_^–^ by CPN-3. Condition: [Tc/Re]_initial_ = 28 ppm and *M*_sorbent_/*V*_solution_ = 1 g/L. (c) Comparison of the sorption capacity and distribution coefficient of benchmark ^99^TcO_4_^–^/ReO_4_^–^ sorbents. (d) Effect of excess competing NO_3_^–^ anions on the ReO_4_^–^ uptake by CPN-3. Condition: [Re]_initial_ = 28 ppm, *M*_sorbent_/*V*_solution_ = 1 g/L, and contact time = 2 h. (e) Comparison of the selectivity toward ReO_4_^–^ by various sorbents in the presence of 100-fold excess of NO_3_^–^. (f) Effect of excess competing SO_4_^2–^ anions on ReO_4_^–^ uptake by CPN-3. Condition: [Re]_initial_ = 28 ppm, *M*_sorbent_/*V*_solution_ = 1 g/L, and contact time = 2 h. (g) Comparison of the selectivity toward ReO_4_^–^ by various sorbents in the presence of 1000-fold excess of SO_4_^2–^.

Considering that sorption capacity is predominantly influenced by anion density, we conducted tests to evaluate the sorption capacities of these materials toward ReO_4_^–^. ReO_4_^–^ serves as an ideal nonradioactive surrogate for the highly radioactive and rare ^99^TcO_4_^–^ due to their similar structure, charge density, and water solubility. The quantities of absorbed ReO_4_^–^ on the materials were determined at the equilibrium state by varying the initial concentrations in the range of 10 to 300 ppm, using a material-to-solution ratio of 0.2 mg/mL. To ensure that the ion exchange had reached equilibrium, a reaction time of 12 h was employed. Figure 3a demonstrates that the sorption of ReO_4_^–^ by all samples exhibits a rapid increase at low concentrations, followed by a notable slowdown as the sorption capacity of the adsorbent is approached. The Langmuir isotherm model provides the best fit for all these isotherms, with high correlation coefficients (>0.97), indicating a monolayer sorption mechanism (Figures S19–21, Table S2). Among the tested materials, CPN-3 demonstrates the highest maximum sorption capacity toward ReO_4_^–^, reaching 1282 mg/g. This surpasses the sorption capacities of CPN-1 and CPN-2, which are 549 and 917 mg/g, respectively. The sorption capacities of these CPNs exhibit the following order: CPN-1 < CPN-2 < CPN-3. This observed trend aligns with the theoretical Cl^–^ densities present in these materials. Also, the sorption capacity of CPN-3 is higher than the benchmark ReO_4_^–^ sorbents with high sorption capacity including SCU-CPN-1 (876 mg/g),^36^ TbDa-COF (952 mg/g),^26^ CPN-tpm (1133 mg/g),^35^ PS-COF-1 (1262 mg/g),^27^ and TFAM-BDNP (998 mg/g)^30^ (Table S4). The superhigh sorption capacity of CPN-3 can effectively prevent the generation of secondary waste and improve efficiency. Given the greatly enhanced performances with the increase of the anion concentration, we establish that the high density of the ion-exchange sites on the polymer backbone is the fundamental contributor to the high working capacity of CPN-3, and the mechanism for uptake of ReO_4_^–^ is thus believed to be predominantly ion exchange.

### Sorption Kinetics Analysis

Subsequently, we conducted experiments to investigate the sorption kinetics of the three CPNs toward ReO_4_^–^. Initially, 20 mg of each CPN was immersed in 100 mL of aqueous solution containing ReO_4_^–^ with a concentration of 28 ppm. As illustrated in Figures S22–24, all three materials reached sorption equilibrium in approximately 15 min. Interestingly, CPN-3 exhibited an outstanding removal efficiency of ReO_4_^–^, surpassing 99.9%. This value is significantly higher than the removal rates observed for CPN-1 (90.0%) and CPN-2 (91.0%). These findings provide further evidence supporting the potential of enhancing the ^99^TcO_4_^–^/ReO_4_^–^ adsorption performance by improving the anion density in the system. The sorption data can be accurately described by the pseudo-second-order model, with CPN-3 exhibiting a *k*_2_ value of 4.10 × 10^–2^ g mg^–1^ min^–1^. The value surpasses the respective *k*_2_ values of 3.98 × 10^–3^ g mg^–1^ min^–1^ for CPN-1 and 5.36 × 10^–3^ g mg^–1^ min^–1^ for CPN-2 (Figures S22–24 and Table S4). The calculated distribution coefficient (*K*_d_) of CPN-3 is determined to be 7.0 × 10^7^ mL/g. To the best of our knowledge, this value is only smaller than that of 3DCOF-*g*-BBPPh_3_Cl (1.0 × 10^8^ mL/g)^46^ and larger than the other benchmark ^99^TcO_4_^–^/ReO_4_^–^ sorbents such as SCU-CPN-4 (1.5 × 10^7^ mL/g),^42^ SCU-102 (5.6 × 10^5^ mL/g),^17^ SCU-100 (1.9 × 10^5^ mL/g),^15^ SCU-COF-1 (3.9 × 10^5^ mL/g),^32^ and VBCOP (4.0 × 10^5^ mL/g)^47^ (Figure 3c, Table S4). With its high *K*_d_ value indicating excellent binding affinity, CPN-3 demonstrates great potential as an effective adsorbent for the removal of ^99^TcO_4_^–^. Considering the rapid sorption kinetics observed for CPN-3 with ReO_4_^–^, we proceeded to investigate its sorption behavior toward ^99^TcO_4_^–^. For this purpose, 20 mg of CPN-3 was immersed in a 20 mL aqueous solution containing ^99^TcO_4_^–^ at a concentration of 28 ppm, replicating the conditions employed in previously reported studies.^42^ The concentration of ^99^TcO_4_^–^ in the solution was monitored over time by measuring the time-dependent radioactivity using liquid scintillation counting (LSC). Figure 3b illustrates that CPN-3 rapidly adsorbed ^99^TcO_4_^–^ and achieved sorption equilibrium within just 1 min, with a removal rate exceeding 99.9%. These findings highlight the exceptional efficiency of CPN-3 in removing ^99^TcO_4_^–^. Furthermore, the adsorption kinetics for ReO_4_^–^ by CPN-3 exhibited the same trend as that of ^99^TcO_4_^–^ under the same conditions, confirming the feasibility of using ReO_4_^–^ as a surrogate (Figure 3b). The sorption kinetics of CPN-3 is truly remarkable, as it is comparable to the benchmark SCU-CPN-1^36^ and SCU-CPN-4^42^ and significantly faster than most of the reported sorbents (Table S4). For example, commercially available resins such as Purolite A532E and A530E were reported to require as long as 120 min to reach sorption equilibrium,^14^ while the removal rate of inorganic material NDTB-1 was only 72% after 36 h of sorption.^12,13^ Even for crystalline cationic MOFs with uniform pore channels like SCU-103^48^ and SCU-101,^19^ the sorption equilibrium was achieved in a minimum of 5–10 min. The remarkable sorption kinetics of CPN-3 makes it particularly appealing for the emergency management of high-level radioactive waste. Its ability to rapidly adsorb ^99^TcO_4_^–^ enables a swift emergency response, effectively reducing the potential risks associated with accidental leakage. This advantage underscores the potential of CPN-3 in addressing urgent situations and enhancing the overall safety and security measures in place for handling high-level radioactive materials.

### Sorption Selectivity

Given the significant presence of competing anions in HLW streams, particularly NO_3_^–^ and SO_4_^2–^, which typically exist over 100–6000 times, we conducted an evaluation of the sorption selectivity of CPN-3 for ReO_4_^–^. Figure 3d demonstrates that the removal efficiencies of CPN-3 toward ReO_4_^–^ are minimally affected at a molar ratio of 1:1 (NO_3_^–^:ReO_4_^–^). Even when NO_3_^–^ is present in 100-fold excess, the removal percentage of ReO_4_^–^ remains remarkably high at 95.0%. This performance exceeds or is on par with that of most reported sorbents, including SCU-CPN-4 (91.5%),^42^ SCU-CPN-1 (91.0%),^36^ SCU-101 (54.4%),^19^ SCU-100 (73.0%),^15^ SCU-CPN-2 (79.0%),^49^ and SCU-103 (88.0%)^48^ (Figure 3e). Remarkably, even in the presence of the highly competitive SO_4_^2–^ anion with its high charge density, CPN-3 exhibits consistent removal rates toward ReO_4_^–^ (>99.9%) even when the amount of SO_4_^2–^ is in a 1000-fold excess (Figure 3f). In comparison, other sorbents such as SCU-CPN-4,^42^ SCU-CPN-2,^49^ SCU-CPN-1,^36^ SCU-101,^19^ SCU-103,^48^ and SCU-100^15^ achieve removal rates of 98.7, 29.7, 70, 83, 89.2, and 73%, respectively, under the same conditions (Figure 3g). The exceptional selectivity of CPN-3 is believed to arise from the presence of alkyl chains, resulting from the free-radical polymerization of carbon–carbon double bonds. These alkyl chains contribute to an increased overall hydrophobicity of the framework, thereby enhancing the affinity for less hydrophilic species such as ^99^TcO_4_^–^ and ReO_4_^–^. The remarkable properties of CPN-3 make it a promising candidate for the remediation of ^99^TcO_4_^–^ in HLW streams, which are characterized by high ionic strengths.

### Sorption after Treatment under High-Alkaline and Radiation Conditions

**Figure 4:**
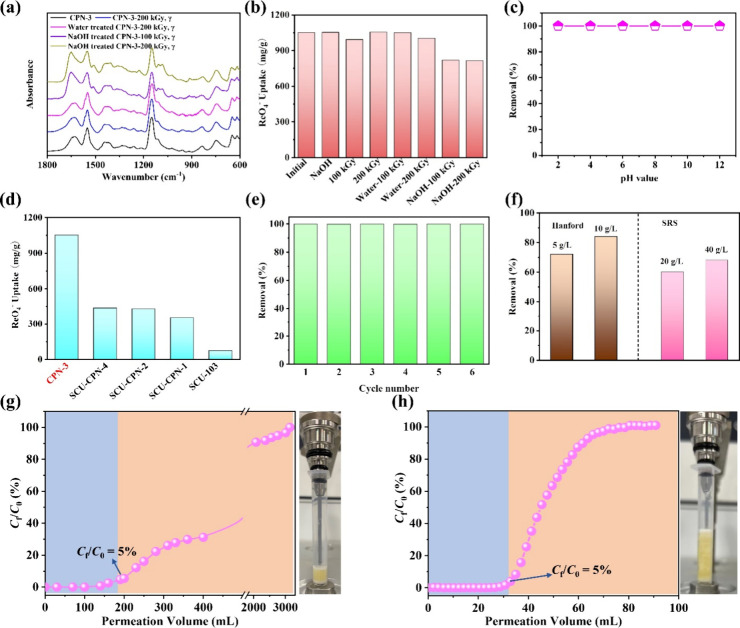
(a) FT-IR spectra of CPN-3, water-treated, and NaOH aqueous solution-treated CPN-3 before and after being irradiated by γ-rays. (b) Sorption capacity of CPN-3 or water/NaOH aqueous solution-treated CPN-3 toward ReO_4_^–^ before and after being irradiated by γ-rays. Condition: [Re]_initial_ = 1000 ppm, *M*_sorbent_/*V*_solution_ = 1 g/L, and contact time = 2 h. (d) Comparison of the sorption capacity of ReO_4_^–^ by various sorbents after being exposed to 1 M NaOH solution for 24 h. Condition: *M*_sorbent_/*V*_solution_ = 1 g/L. (e) Reusability of CPN-3 for the sorption of ReO_4_^–^. (f) The removal of ^99^TcO_4_^–^ from simulated Hanford LAW and SRS HAW by CPN-3 with different solid/liquid ratios. (g) Dynamic sorption column analysis of CPN-3. Condition: [Re]_initial_ = 28 ppm, *m*_sorbent_ = 40 mg, and flow rate = 4.0 mL/min. (h) Dynamic sorption column analysis of CPN-3 under simulated Hanford LAW waste stream. Condition: [Re] = 14 ppm, *m*_sorbent_ = 200 mg, and flow rate = 2.0 mL/min.

Considering the challenging conditions present in HLW streams, which involve strong ionizing fields (β, γ, neutron irradiations) and highly alkaline environments, sorbents must possess resistance to radiation and alkaline effects to be practically applicable. However, certain sorbents, like the commercially available Purolite A530E resin, despite exhibiting good sorption performance for the removal of TcO_4_^–^, suffer from poor radiation stability.^36^ This inherent drawback significantly limits its practical use for the removal of ^99^TcO_4_^–^ from HLW streams. Consequently, it is necessary to conduct a systematic assessment of CPN-3’s adsorption capabilities toward ReO_4_^–^ after treatment under high-alkalinity and irradiation conditions. Before initiating the adsorption experiments, the stability of CPN-3 under these harsh conditions was characterized using FT-IR spectroscopy. As depicted in Figure 4a, minimal changes were observed in the FT-IR spectra of CPN-3 even after treatment with 1 M NaOH aqueous solutions and subsequent exposure to 200 kGy of γ-radiation, thus confirming the structural integrity of CPN-3 under such severe conditions. We then conducted experiments to evaluate the ReO_4_^–^ removal performance of CPN-3 after being treated with high-alkaline and radioactive conditions step by step. First, we immersed CPN-3 in water or NaOH aqueous solutions (1 M) and stirred the mixture for 24 h. The resulting material was then centrifuged and dried in a 60 °C oven for 4 h to obtain the water or NaOH-treated CPN-3. Then, the pristine, water-treated, and NaOH-treated CPN-3 were exposed to 100 and 200 kGy of γ-radiation, respectively, to obtain the radiation exposure samples. Both these samples were used for ReO_4_^–^ sorption experiments. Figure 4b illustrates that the ReO_4_^–^ adsorption capacity of NaOH-treated CPN-3 (1052 mg/g) is comparable to that of the pristine sample (1051 mg/g), indicating the excellent stability of CPN-3 under highly alkaline conditions. It is worth noting that most reported ReO_4_^–^ sorbents exhibit decreased sorption capacities under the same alkaline conditions. For instance, SCU-CPN-1, SCU-CPN-2, and SCU-103 experience significant reductions in sorption capacities, dropping from 953, 1308, and 328 mg/g to 350, 429, and 73 mg/g, respectively.^42^ This significant decrease highlights the instability of these materials when exposed to highly alkaline conditions. Furthermore, the sorption capacity of NaOH-treated CPN-3 (1052 mg/g) surpasses the record-holding SCU-CPN-4 (∼437 mg/g) by 2.4 times, establishing a new record in ReO_4_^–^ sorption capacity after treatment under high-alkaline conditions (Figure 4d).^42^ In addition, the sorption capacity of water-treated CPN-3 after exposure under 200 kGy of γ-radiation is almost the same as that of the pristine sample, demonstrating the excellent stabilities of these samples. For the samples treated with 1 M NaOH aqueous solutions and γ-radiation, a 22% decrease in the sorption capacities was observed, suggesting that part of the samples was destroyed under such conditions. However, the sorption capacity is still as high as 817 mg/g (Figure 4b). In addition, CPN-3 demonstrated a negligible decrease in the removal efficiency within a wide pH range of 2 to 12 (Figure 4c). The remarkable stability of CPN-3 under highly radioactive and alkaline conditions is attributed to the presence of alkyl chains formed through the free-radical polymerization of carbon–carbon double bonds. These alkyl chains play a crucial role in protecting the imidazole groups from ring-opening reactions caused by the attack of OH^–^ ions, thereby enhancing the overall stability of the framework under such challenging conditions. The remarkable achievement further underscores the exceptional performance of CPN-3 as a highly stable and efficient sorbent for TcO_4_^–^ removal, even in challenging high-alkaline and radioactive environments.

### Recyclability Investigation

The reusability of sorbents plays a vital role in their practical application, and to assess the reusability of CPN-3, a 1 M NaCl solution was utilized to elute ReO_4_^–^ from the sorbents due to the strong competition of Cl^–^ with ReO_4_^–^ in anion exchange. Figure 4e illustrates that, during the initial sorption cycle, over 99.9% of ReO_4_^–^ was effectively removed when 20 mg of CPN-3 was immersed in a 20 mL ReO_4_^–^ solution (28 ppm) and stirred for 2 h. Subsequently, washing CPN-3 with a 1 M NaCl solution successfully stripped off over 99% of the captured ReO_4_^–^. The regenerated CPN-3 demonstrated exceptional recyclability, maintaining a removal efficiency exceeding 99% even after six cycles. This outstanding reusability highlights CPN-3’s capability for efficient sequestration of ^99^TcO_4_^–^/ReO_4_^–^ in nuclear waste management.

### Dynamic Separation Experiments

To evaluate the sorption performance of CPN-3 in practical applications, dynamic sorption experiments were conducted by using CPN-3 as a filler. Considering the exceptional sorption capacity and rapid sorption rate of CPN-3 for ReO_4_^–^, we utilized 40 mg samples to conduct this breakthrough experiment. A prepared ReO_4_^–^ aqueous solution with a concentration of 28 ppm was introduced into the column at a flow rate of 4.0 mL/min. As shown in Figure 4g, a significant portion of ReO_4_^–^ was removed, with a residual concentration (*C*_f_/*C*_0_) less than 5% after passing through 200 mL of the waste solution. This indicates that CPN-3 can treat a mass of ReO_4_^–^ solution approximately 5000 times its own mass. The high removal efficiency observed can be attributed to the ultrafast sorption kinetics of CPN-3. The rapid sorption rate allows for an impressive removal efficiency even in a flowing solution. As the solution flowed through the column, the concentration of ReO_4_^–^ in the effluent gradually increased. The dynamic separation results highlight the excellent practical application potential of CPN-3 in the treatment of radioactive waste containing ^99^TcO_4_^–^.

### Removal from Simulated Nuclear Wastes

The rapid kinetics, high adsorption capacity, and exceptional selectivity of CPN-3 prompted us to explore its performance in addressing legacy nuclear waste challenges. Previous evaluations of Hanford tank waste inventories have indicated the necessity of significantly reducing technetium levels to prepare immobilized low-activity waste (ILAW) glass that fulfills performance assessment requirements. To investigate the removal of ^99^TcO_4_^–^ for this purpose, we conducted experiments using a simulated Hanford LAW wastewater solution. The concentrations of NO_3_^–^ (5.96 × 10^–2^ M), NO_2_^–^ (3.03 × 10^–3^ M), and Cl^–^ (6.93 × 10^–2^ M) were intentionally maintained at over 300 times higher levels than that of ^99^TcO_4_^–^ (1.94 × 10^–4^ M), creating a formidable challenge for selective ^99^TcO_4_^–^ removal (Table S5). Notably, CPN-3 exhibited exceptional performance, removing over 72 and 84% of ^99^TcO_4_^–^ at solid/liquid ratios of 5 and 10 g/L, respectively, surpassing the capabilities of SCU-CPN-2 (67%, 5 g/L),^49^ NDTB-1 (13%, 5 g/L),^13^ SCU-COF-1 (62.8, 10 g/L),^32^ and SCU-101 (75.2%, 10 g/L)^19^ (Figure 4f and Table S7). Furthermore, column sorption tests using a simulated Hanford LAW stream demonstrated that an ion-exchange chromatographic column packed with 200 mg of CPN-3 effectively removed nearly all of the ReO_4_^–^ from the initial 32 mL of the waste solution, indicating high removal efficiency (Figure 4h). To expand the potential application of CPN-3 in nuclear waste remediation, we tested its capability for decontaminating ^99^TcO_4_^–^ at Savannah River Sites (SRS). The SRS HLW waste is an extremely competitive and superalkaline stream, with significantly higher amounts of OH^–^, NO_3_^–^, SO_4_^2–^, and NO_2_^–^ compared to TcO_4_^–^. Specifically, the amounts of OH^–^, NO_3_^–^, SO_4_^2–^, and NO_2_^–^ in the waste are over TcO_4_^–^ by factors of 16 788, 32 819, 6576, and 1691, respectively (Table S6). In simulated SRS waste, the removal of TcO_4_^–^ by CPN-3 reached 68% at a solid/liquid ratio of 40 g/L, lower than those of SCU-CPN-4 (94.3%, 20 g/L),^42^ Ag-TPPE (90.0%, 10 g/L),^50^ and SCU-103 (90.0%, 40 g/L)^48^ (Figure 4f, Table S7). It is important to emphasize that dealing with Hanford LAW and SRS HAW waste requires a substantial quantity of adsorbents, often measured in tons, making the cost of materials and scalability crucial factors that are currently widely overlooked. The laboratory synthesis cost of CPN-3 is estimated to be 3 USD/g, and its simple synthesis steps allow for easy upscaling, leading to further cost reduction. In contrast, other highly efficient ^99^TcO_4_^–^/ReO_4_^–^ adsorbents mentioned in the literature, like Ag-TPPE, SCU-CPN-4, and SCU-103, suffer from complex organic ligand/monomer synthesis processes, expensive reaction materials, and challenging large-scale production, making them impractical for real-world applications. With exceptional structural stability, moderate removal efficiency, and low material cost, CPN-3 emerges as an attractive option for direct removal of ^99^TcO_4_^–^ from legacy nuclear waste streams such as Hanford LAW and SRS HAW waste.

### Sorption Mechanism

**Figure 5:**
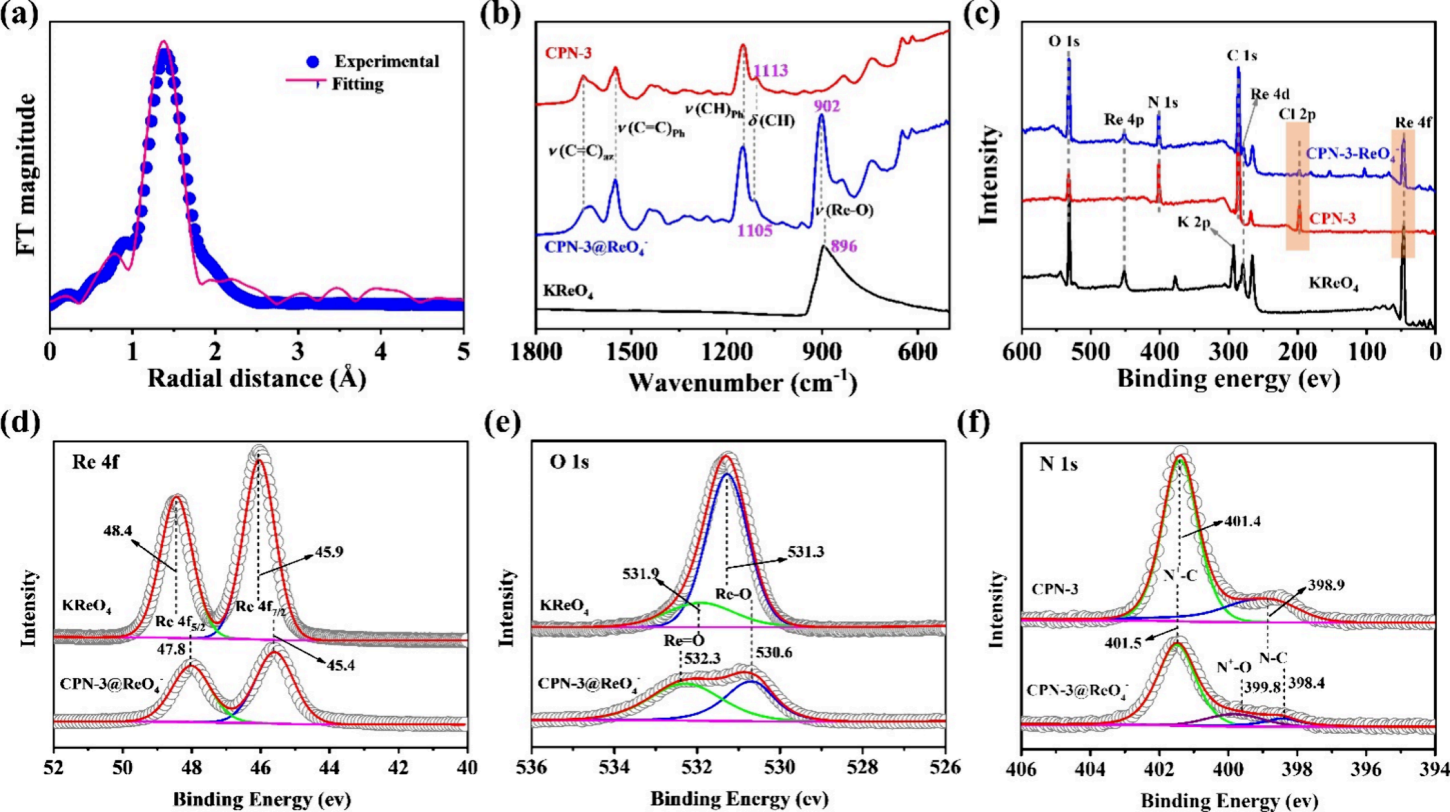
(a) Re L_3_-edge EXAFS fitting curve for CPN-3@ReO_4_^–^. (b) FT-IR spectra of KReO_4_, CPN-3, and CPN-3@ReO_4_^–^. (c) XPS survey spectra of KReO_4_, CPN-3, and CPN-3@ReO_4_^–^. (d) Re 4f XPS spectra for KReO_4_ and CPN-3@ReO_4_^–^. (e) O 1s XPS spectra for KReO_4_ and CPN-3@ReO_4_^–^. (f) N 1s XPS spectra for CPN-3 and CPN-3@ReO_4_^–^.

To gain a deeper understanding of the sorption mechanism and superior performance of CPN-3 toward ^99^TcO_4_^–^/ReO_4_^–^, we conducted a series of examination studies. SEM-EDS analysis of CPN-3 and CPN-3@ReO_4_^–^ revealed a distinct change in the distribution of Cl and Re elements, suggesting a close relationship between the appearance of Re and the disappearance of Cl in the sorbents (Figure S13). Additionally, the consistent morphology of CPN-3 observed before and after the sorption of ReO_4_^–^ indicated that the polymeric networks of the sorbents remained unchanged (Figures S12 and 13). The electronic structure of adsorbed ReO_4_^–^ and the binding sites on CPN-3 were further investigated by using L_3_-edge X-ray absorption fine spectroscopy (XAFS). The Re L_3_-edge X-ray absorption near-edge structure (XANES) spectra revealed that the absorption edge position for ReO_4_^–^ adsorbed on CPN-3 was 10 541 eV, which closely matched that of NH_4_ReO_4_ (10 540 eV),^51^ indicating that the presence of Re in the +7 oxidation state (Figure S25). In the corresponding Re L_3_-edge R-space-extended XAFS (EXAFS) spectra, a single prominent peak at approximately 1.35 Å (not phase shift corrected) was observed. This peak could be accurately fitted using a Re–O scattering path, indicating that the adsorption of ReO_4_^–^ occurred in the form of the ReO_4_^–^ anion (Figure 5a). The FT-IR spectra of CPN-3, CPN-3@ReO_4_^–^, and KReO_4_ are presented in Figure 5b. It is evident from the spectra that the presence of ReO_4_^–^ adsorption is characterized by a well-defined band at 902 cm^–1^ in the spectrum of CPN-3@ReO_4_^–^, which is notably absent in the spectrum of CPN-3. This distinctive band can be attributed to the symmetric Re–O stretching vibration, as documented by previous studies.^52^ In comparison to the spectrum of KReO_4_, the band center of the ν(ReO_4_) peak exhibits a noticeable upward (blue-) shift of +6 cm^–1^. Additionally, the line width of the ν(ReO_4_) vibration in CPN-3@ReO_4_^–^ is considerably reduced, providing a clear indication that the ReO_4_^–^ cluster is confined to well-distributed and localized binding sites within the network. Careful examination of CPN-3 spectra before and after adsorbing ReO_4_^–^ reveals subtle changes to its phonon modes of the azole ring, e.g., intensity of the ν(C=C) mode diminished and the δ(CH) mode blue-shifted by 8 cm^–1^ (Figures 5b, S6). In contrast, the phenyl ring modes ν(C=C) and δ(CH), which were previously shown to be sensitive to the chemical environment change, remain in the same position.^53,54^ All of these findings point to the fact that ReO_4_^–^ interacts primarily with the azole ring of CPN-3. XPS analysis is used to further analyze the ReO_4_^–^ adsorption site on CPN-3. The observation of the Re 4f signal and the attenuation of Cl 2p peak in the survey spectrum of CPN-3@ReO_4_^–^ further demonstrate that the Re (VII) oxidation state exists before and after adsorption and the adsorption of ReO_4_^–^ followed by anion exchange (Figure 5c). The peak corresponding to Re 4f_7/2_ undergoes a shift from 45.9 eV in the case of ReO_4_^–^ to 45.4 eV in the case of CPN-3@ReO_4_^–^, while a similar phenomenon is observed in the shift of the O 1s peak attributed to Re–O^–^ from 531.3 eV for ReO_4_^–^ to 530.6 eV for CPN-3@ReO_4_^–^ (Figure 5d,e). These shifts in peak positions suggest that the electron density of ReO_4_^–^ experiences a slight increase following its interaction with the CPN-3 framework, a change that is likely facilitated by the presence of imidazolium units in the pores of CPN-3, which can provide positive charges to counterbalance the negatively charged ReO_4_^–^ species. A noteworthy observation in the XPS spectrum of CPN-3@ReO_4_^–^ is the emergence of a new peak at 399.8 eV, corresponding to the N^+^–O^–^ species, providing further confirmation that the primary adsorption sites are the N^+^ moieties within the imidazolium units (Figure 5f). Similar phenomena have been observed in a published work.^55^

**Figure 6:**
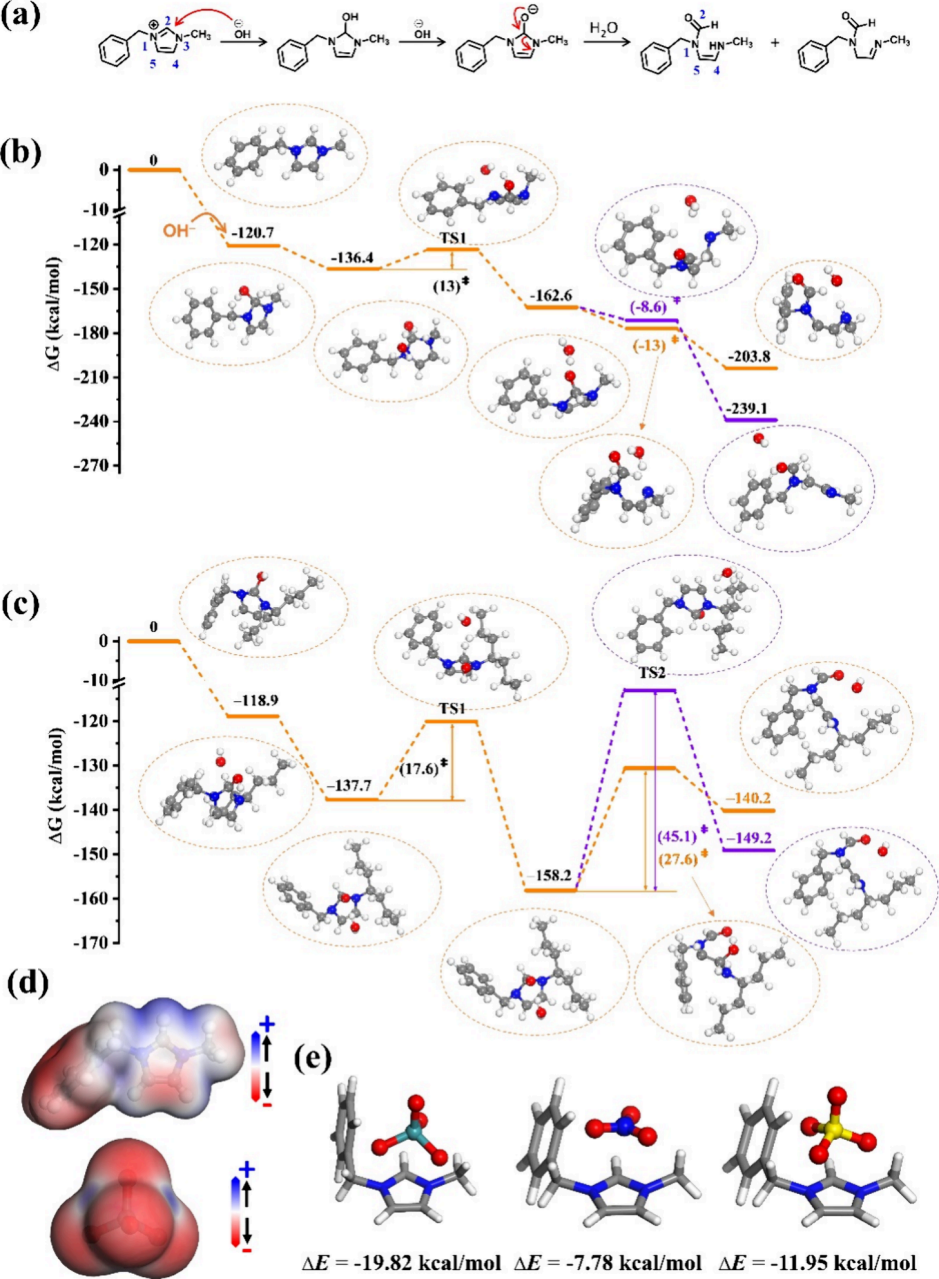
(a) Degradation of the imidazolium unit by the ring-opening mechanism. The DFT calculated ring-opening mechanism and the corresponding Gibbs energy profiles of Fragment 1 (b) and Fragment 2 (c) by the attack of OH^–^ in alkaline solutions. (d) ESP distribution on the electron density surface (isodensity = 0.001 au) of the M^+^ fragment (top) and ^99^TcO_4_^–^ (bottom). (e) Optimized structures of M^+^TcO_4_^–^ (left), M^+^NO_3_^–^ (middle), and M^+^SO_4_^2–^ (right). Δ*E* represents the binding energy.

Density functional theory (DFT) calculations were further conducted to investigate the mechanisms behind the remarkable alkaline stability of CPN-3 and its superior separation capability toward ^99^TcO_4_^–^. In alkaline environments, the nucleophilic reagent OH^–^ is known to preferentially attack the C2 position of imidazolium ions, resulting in the degradation of the imidazolium ring through nucleophilic addition, ring-opening, and rearrangement reactions.^37−40^Figure 6a illustrates the widely accepted ring-opening pathway of the imidazolium ring under alkaline conditions, which can be applied to describe the degradation of imidazolium-based CPNs. Two fragments, labeled Fragments 1 and 2, were selected to simulate the properties of two specific sorbents. Fragment 1 represents SCU-CPN-1, a highly efficient ^99^TcO_4_^–^ sorbent that is known to be unstable in alkaline conditions.^42^ On the other hand, Fragment 2 represents CPN-3, the sorbent reported in this study, which exhibits excellent stability in alkaline solutions. Figure 6b,c illustrates the ring-opening reaction pathway of the imidazole unit for Fragments 1 and 2, respectively. The adsorption of OH^–^ onto the imidazole ring in both Fragment 1 and Fragment 2 is an exothermic process, releasing energy of 120.7 and 118.9 kcal/mol, respectively. Subsequently, another free OH^–^ species approaches and reacts with OH^–^ adsorbed on the imidazole ring, leading to the formation of a water molecule. In the case of Fragment 1, this reaction exhibits a Gibbs energy barrier of 13 kcal/mol, whereas for Fragment 2, the barrier is higher at 17.6 kcal/mol, suggesting that the OH^–^ attack to form H_2_O is more kinetically demanding for Fragment 2. The ring-opening reaction proceeds with the assistance of water molecules. In the case of Fragment 1, the calculated Gibbs free reaction energies are −8.6 and −13.0 kcal/mol for the two plausible ring-opening scenarios, and no transition state is found. The ring-opening process is continuous, exothermic, and spontaneous. This indicates that imidazole cations in Fragment 1 exhibit high instability in alkaline solutions and are prone to undergoing ring-opening reactions, which aligns with the experimental observations. In contrast, for Fragment 2, the Gibbs free energies of activation for both types of ring-opening reactions are significantly high, measuring 27.6 and 45.1 kcal/mol, respectively, as depicted in Figure 6c. Additionally, our calculations reveal that the ring-opening steps in Fragment 2 are endergonic, with Gibbs free reaction energies of +9.0 and +18.0 kcal/mol. Therefore, the presence of the bulk alkyl chain in Fragment 2 thermodynamically and kinetically inhibits the ring-opening process of imidazole rings, thus providing Fragment 2 with exceptional stability in alkaline solutions. These findings align with the experimental observations.

DFT calculations were then conducted to reveal the excellent selectivity of CPN-3 toward ^99^TcO_4_^–^ over other ions such as NO_3_^–^ and SO_4_^2–^. As mentioned above, the imidazolium unit serves as the primary sorption site, and M^+^ (representing the cation) was chosen as the model for the calculations. Initially, we examined the electrostatic potential (ESP) of M^+^ and studied that of ^99^TcO_4_^–^ for comparison purposes. It is not surprising that the electron density van der Waals surface near the imidazole rings of M^+^ exhibited a relatively concentrated positive ESP distribution (Figure 6d top). The electron density near the oxygen atoms of ^99^TcO_4_^–^ displayed a relatively concentrated negative ESP distribution (Figure 6d bottom). This mutual attraction between the positive and negative charges is considered to be the intrinsic reason for the excellent adsorption performance of CPN-3 toward ^99^TcO_4_^–^. We then optimized the structures of M^+^NO_3_^–^, M^+^SO_4_^2–^, and M^+^TcO_4_^–^ and determined their respective binding energy (Δ*E*) values. The results, depicted in Figure 6e, reveal that the primary adsorption sites for all three anions are located in the proximity of the positively charged imidazolium unit, consistent with experimental observations. Notably, despite exhibiting similar sorption sites on the M^+^ fragment, the corresponding Δ*E* values show distinct differences. Specifically, the calculated Δ*H* value for M^+^TcO_4_^–^ is determined to be −19.82 kcal/mol, surpassing that of M^+^NO_3_^–^ (−7.78 kcal/mol) and M^+^SO_4_^2–^ (−11.95 kcal/mol). This discrepancy indicates that ^99^TcO_4_^–^ displays a greater energetic favorability in binding to the M^+^ fragment compared to NO_3_^–^ and SO_4_^2–^, elucidating the enhanced selectivity of CPN-3 toward ^99^TcO_4_^–^.

## Conclusions

In summary, a cationic polymeric nanotrap called CPN-3 was designed and synthesized to address the challenges associated with alkaline nuclear waste management. CPN-3 was meticulously engineered by maximizing the density of counteranions and incorporating a superhydrophobic alkyl chain at the N3 position of the key imidazolium moiety. This sophisticated design approach resulted in CPN-3 possessing exceptional alkaline resistance, attributed to the presence of the alkyl chain, as well as enhanced hydrophobicity, leading to improved selectivity toward ^99^TcO_4_^–^. The studies conducted on CPN-3 revealed its outstanding sorption properties, which encompassed fast sorption kinetics, high sorption capacity, excellent sorption selectivity, complete reusability, and remarkable sorption performance even under highly alkaline aqueous solutions. These notable features make CPN-3 a highly promising candidate for the efficient removal of ^99^TcO_4_^–^ from alkaline nuclear waste, thereby offering a new avenue for addressing the persistent challenges encountered in the CARBEX process and alkaline nuclear waste management.

## Supporting Information Available

Full details for the material characterization, synthesis of monomers, synthesis of CPNs, calculation of the theoretical Cl^–^ densities, alkaline stability exploration, alkaline and irradiation stability exploration, sorption isotherm investigations, batch experiments, sorption kinetics study, computational methods, supplementary figures, supplementary tables, and References (PDF)

## References

[ref1] Murray R.; Holbert K. E. Nuclear energy: An introduction to the concepts, systems, and applications of nuclear processes, 7th ed.; Elsevier, 2014.

[ref2] Taylor R. Reprocessing and recycling of spent nuclear fuel; Elsevier, 2015.

[ref3] Herbst R.; Baron P.; Nilsson M. Standard and advanced separation: PUREX processes for nuclear fuel reprocessing. Advanced separation techniques for nuclear fuel reprocessing and radioactive waste treatment 2011, 141–175. 10.1533/9780857092274.2.141.

[ref4] Smirnov I.; Karavan M.; Logunov M.; Tananaev I.; Myasoedov B. Extraction of radionuclides from alkaline and carbonate media. Radiochemistry 2018, 60, 470–487. 10.1134/S1066362218050028.

[ref5] Smirnov I. V.; Stepanova E. S.; Tyupina M. Y.; Ivenskaya N. M.; Tananaev I. G.; Zaripov S. R.; Kleshnina S. R.; Solov’eva S. E.; Antipin I. S. Effect of ionizing radiation on the extraction of Am(III) with p-tert-butylthiacalix[4]arene from alkaline carbonate solutions. Radiochemistry 2017, 59 (4), 365–371. 10.1134/S1066362217040087.

[ref6] Page J. S.; Reynolds J. G.; Ely T. M.; Cooke G. A. Development of a carbonate crust on alkaline nuclear waste sludge at the Hanford site. J. Hazard. Mater. 2018, 342, 375–382. 10.1016/j.jhazmat.2017.08.033. 28850915

[ref7] Beals D. M.; Hayes D. W. Technetium-99, iodine-129 and tritium in the waters of the Savannah River Site. Sci. Total Environ. 1995, 173–174, 101–15. 10.1016/0048-9697(95)04769-7. 8560219

[ref8] Banerjee D.; Kim D.; Schweiger M. J.; Kruger A. A.; Thallapally P. K. Removal of TcO_4_^–^ ions from solution: materials and future outlook. Chem. Soc. Rev. 2016, 45 (10), 2724–2739. 10.1039/C5CS00330J. 26947251

[ref9] Xiao C.; Khayambashi A.; Wang S. Separation and Remediation of ^99^TcO_4_^–^ from Aqueous Solutions. Chem. Mater. 2019, 31 (11), 3863–3877. 10.1021/acs.chemmater.9b00329.

[ref10] Wang Y.; Gao H. Compositional and structural control on anion sorption capability of layered double hydroxides (LDHs). J. Colloid Interface Sci. 2006, 301 (1), 19–26. 10.1016/j.jcis.2006.04.061. 16750215

[ref11] Goulding H. V.; Hulse S. E.; Clegg W.; Harrington R. W.; Playford H. Y.; Walton R. I.; Fogg A. M. Yb_3_O(OH)_6_Cl·2H_2_O: an anion-exchangeable hydroxide with a cationic inorganic framework structure. J. Am. Chem. Soc. 2010, 132 (39), 13618–13620. 10.1021/ja104636x. 20614886

[ref12] Wang S.; Alekseev E. V.; Diwu J.; Casey W. H.; Phillips B. L.; Depmeier W.; Albrecht-Schmitt T. E. NDTB-1: a supertetrahedral cationic framework that removes TcO_4_^–^ from solution. Angew. Chem., Int. Ed. 2010, 49 (6), 1057–1060. 10.1002/anie.200906397. 20039250

[ref13] Wang S.; Yu P.; Purse B. A.; Orta M. J.; Diwu J.; Casey W. H.; Phillips B. L.; Alekseev E. V.; Depmeier W.; Hobbs D. T.; et al. Selectivity, Kinetics, and Efficiency of Reversible Anion Exchange with TcO_4_^–^ in a Supertetrahedral Cationic Framework. Adv. Funct. Mater. 2012, 22 (11), 2241–2250. 10.1002/adfm.201103081.

[ref14] Li J.; Zhu L.; Xiao C.; Chen L.; Chai Z.; Wang S. Efficient uptake of perrhenate/pertechnenate from aqueous solutions by the bifunctional anion-exchange resin. Radiochim. Acta 2018, 106 (7), 581–591. 10.1515/ract-2017-2829.

[ref15] Sheng D.; Zhu L.; Xu C.; Xiao C.; Wang Y.; Wang Y.; Chen L.; Diwu J.; Chen J.; Chai Z.; et al. Efficient and Selective Uptake of TcO_4_^–^ by a Cationic Metal-Organic Framework Material with Open Ag^+^ Sites. Environ. Sci. Technol. 2017, 51 (6), 3471–3479. 10.1021/acs.est.7b00339. 28211267

[ref16] Zhu L.; Xiao C.; Dai X.; Li J.; Gui D.; Sheng D.; Chen L.; Zhou R.; Chai Z.; Albrecht-Schmitt T. E.; et al. Exceptional Perrhenate/Pertechnetate Uptake and Subsequent Immobilization by a Low-Dimensional Cationic Coordination Polymer: Overcoming the Hofmeister Bias Selectivity. Environ. Sci. Technol. Lett. 2017, 4 (7), 316–322. 10.1021/acs.estlett.7b00165.

[ref17] Sheng D.; Zhu L.; Dai X.; Xu C.; Li P.; Pearce C. I.; Xiao C.; Chen J.; Zhou R.; Duan T.; et al. Successful Decontamination of ^99^TcO_4_^–^ in Groundwater at Legacy Nuclear Sites by a Cationic Metal-Organic Framework with Hydrophobic Pockets. Angew. Chem., Int. Ed. 2019, 58 (15), 4968–4972. 10.1002/anie.201814640. 30761705

[ref18] Soe E.; Ehlke B.; Oliver S. R. J. A Cationic Silver Pyrazine Coordination Polymer with High Capacity Anion Uptake from Water. Environ. Sci. Technol. 2019, 53 (13), 7663–7672. 10.1021/acs.est.9b01633. 31174421

[ref19] Zhu L.; Sheng D.; Xu C.; Dai X.; Silver M. A.; Li J.; Li P.; Wang Y.; Wang Y.; Chen L.; et al. Identifying the Recognition Site for Selective Trapping of ^99^TcO_4_^–^ in a Hydrolytically Stable and Radiation Resistant Cationic Metal-Organic Framework. J. Am. Chem. Soc. 2017, 139 (42), 14873–14876. 10.1021/jacs.7b08632. 28985681

[ref20] Song Y.; Phipps J.; Zhu C.; Ma S. Porous Materials for Water Purification. Angew. Chem., Int. Ed. 2023, 62 (11), e202216724 10.1002/anie.202216724. 36538551

[ref21] Skorjanc T.; Shetty D.; Trabolsi A. Pollutant removal with organic macrocycle-based covalent organic polymers and frameworks. Chem. 2021, 7 (4), 882–918. 10.1016/j.chempr.2021.01.002.

[ref22] Abney C. W.; Mayes R. T.; Saito T.; Dai S. Materials for the Recovery of Uranium from Seawater. Chem. Rev. 2017, 117 (23), 13935–14013. 10.1021/acs.chemrev.7b00355. 29165997

[ref23] Das S.; Heasman P.; Ben T.; Qiu S. Porous Organic Materials: Strategic Design and Structure-Function Correlation. Chem. Rev. 2017, 117 (3), 1515–1563. 10.1021/acs.chemrev.6b00439. 28035812

[ref24] Geng K.; He T.; Liu R.; Dalapati S.; Tan K. T.; Li Z.; Tao S.; Gong Y.; Jiang Q.; Jiang D. Covalent Organic Frameworks: Design, Synthesis, and Functions. Chem. Rev. 2020, 120 (16), 8814–8933. 10.1021/acs.chemrev.9b00550. 31967791

[ref25] Tian Y.; Zhu G. Porous Aromatic Frameworks (PAFs). Chem. Rev. 2020, 120 (16), 8934–8986. 10.1021/acs.chemrev.9b00687. 32101403

[ref26] Wang Y.; Xie M.; Lan J.; Yuan L.; Yu J.; Li J.; Peng J.; Chai Z.; Gibson J. K.; Zhai M.; et al. Radiation Controllable Synthesis of Robust Covalent Organic Framework Conjugates for Efficient Dynamic Column Extraction of ^99^TcO_4_^–^. Chem. 2020, 6 (10), 2796–2809. 10.1016/j.chempr.2020.08.005.

[ref27] Hao M.; Chen Z.; Yang H.; Waterhouse G. I. N.; Ma S.; Wang X. Pyridinium salt-based covalent organic framework with well-defined nanochannels for efficient and selective capture of aqueous ^99^TcO_4_^–^. Sci. Bull. 2022, 67 (9), 924–932. 10.1016/j.scib.2022.02.012. 36546027

[ref28] Cui W. R.; Xu W.; Chen Y. R.; Liu K.; Qiu W. B.; Li Y.; Qiu J. D. Olefin-linked cationic covalent organic frameworks for efficient extraction of ReO_4_^–^/^99^TcO_4_^–^. J. Hazard. Mater. 2023, 446, 130603 10.1016/j.jhazmat.2022.130603. 36580784

[ref29] Peng H.; Jiang B.; Li F.; Gong J.; Zhang Y.; Yang M.; Liu N.; Ma L. Single-Crystal-Structure Directed Predesign of Cationic Covalent Organic Polymers for Rapidly Capturing ^99^TcO_4_^–^. Chem. Mater. 2023, 35 (6), 2531–2540. 10.1021/acs.chemmater.2c03812.

[ref30] Chen X.-R.; Zhang C.-R.; Jiang W.; Liu X.; Luo Q.-X.; Zhang L.; Liang R.-P.; Qiu J.-D. 3D Viologen-based covalent organic framework for selective and efficient adsorption of ReO_4_^–^/TcO_4_^–^. Sep. Purif. Technol. 2023, 312, 123409 10.1016/j.seppur.2023.123409.

[ref31] Da H. J.; Yang C. X.; Yan X. P. Cationic Covalent Organic Nanosheets for Rapid and Selective Capture of Perrhenate: An Analogue of Radioactive Pertechnetate from Aqueous Solution. Environ. Sci. Technol. 2019, 53 (9), 5212–5220. 10.1021/acs.est.8b06244. 30933484

[ref32] He L.; Liu S.; Chen L.; Dai X.; Li J.; Zhang M.; Ma F.; Zhang C.; Yang Z.; Zhou R.; et al. Mechanism unravelling for ultrafast and selective ^99^TcO_4_^–^ uptake by a radiation-resistant cationic covalent organic framework: a combined radiological experiment and molecular dynamics simulation study. Chem. Sci. 2019, 10 (15), 4293–4305. 10.1039/C9SC00172G. 31057756 PMC6471554

[ref33] Di Z.; Mao Y.; Yuan H.; Zhou Y.; Jin J.; Li C.-P. Covalent Organic Frameworks(COFs) for Sequestration of ^99^TCO_4_^–^. Chem. Res. Chin. Univ. 2022, 38 (2), 290–295. 10.1007/s40242-022-1447-9.

[ref34] Sun Q.; Zhu L.; Aguila B.; Thallapally P. K.; Xu C.; Chen J.; Wang S.; Rogers D.; Ma S. Optimizing radionuclide sequestration in anion nanotraps with record pertechnetate sorption. Nat. Commun. 2019, 10 (1), 1646 10.1038/s41467-019-09630-y. 30967551 PMC6456584

[ref35] Li X.; Li Y.; Wang H.; Niu Z.; He Y.; Jin L.; Wu M.; Wang H.; Chai L.; Al-Enizi A. M.; et al. 3D Cationic Polymeric Network Nanotrap for Efficient Collection of Perrhenate Anion from Wastewater. Small 2021, 17 (20), e2007994 10.1002/smll.202007994. 33749108

[ref36] Li J.; Dai X.; Zhu L.; Xu C.; Zhang D.; Silver M. A.; Li P.; Chen L.; Li Y.; Zuo D.; et al. ^99^TcO_4_^–^ remediation by a cationic polymeric network. Nat. Commun. 2018, 9 (1), 3007 10.1038/s41467-018-05380-5. 30068903 PMC6070552

[ref37] Gottesfeld S.; Dekel D. R.; Page M.; Bae C.; Yan Y.; Zelenay P.; Kim Y. S. Anion exchange membrane fuel cells: Current status and remaining challenges. J. Power Sources 2018, 375, 170–184. 10.1016/j.jpowsour.2017.08.010.

[ref38] Hugar K. M.; Kostalik H. A. t.; Coates G. W. Imidazolium Cations with Exceptional Alkaline Stability: A Systematic Study of Structure-Stability Relationships. J. Am. Chem. Soc. 2015, 137 (27), 8730–8737. 10.1021/jacs.5b02879. 26062959

[ref39] Gu F.; Dong H.; Li Y.; Si Z.; Yan F. Highly Stable N3-Substituted Imidazolium-Based Alkaline Anion Exchange Membranes: Experimental Studies and Theoretical Calculations. Macromolecules 2014, 47 (1), 208–216. 10.1021/ma402334t.

[ref40] Salma U.; Shalahin N. A mini-review on alkaline stability of imidazolium cations and imidazolium-based anion exchange membranes. Results in Materials 2023, 17, 100366 10.1016/j.rinma.2023.100366.

[ref41] Yang X.; Wu W.; Xie Y.; Hao M.; Liu X.; Chen Z.; Yang H.; Waterhouse G. I. N.; Ma S.; Wang X. Modulating Anion Nanotraps via Halogenation for High-Efficiency ^99^TcO_4_^–^/ReO_4_^–^ Removal under Wide-Ranging pH Conditions. Environ. Sci. Technol. 2023, 57 (29), 10870–10881. 10.1021/acs.est.3c02967. 37431600

[ref42] Li J.; Li B.; Shen N.; Chen L.; Guo Q.; Chen L.; He L.; Dai X.; Chai Z.; Wang S. Task-Specific Tailored Cationic Polymeric Network with High Base-Resistance for Unprecedented ^99^TcO_4_^–^ Cleanup from Alkaline Nuclear Waste. ACS Cent. Sci. 2021, 7 (8), 1441–1450. 10.1021/acscentsci.1c00847. 34471688 PMC8393213

[ref43] Suo X.; Cui X.; Yang L.; Xu N.; Huang Y.; He Y.; Dai S.; Xing H. Synthesis of Ionic Ultramicroporous Polymers for Selective Separation of Acetylene from Ethylene. Adv. Mater. 2020, 32 (29), e1907601 10.1002/adma.201907601. 32529690

[ref44] Suo X.; Yu Y.; Qian S.; Zhou L.; Cui X.; Xing H. Tailoring the Pore Size and Chemistry of Ionic Ultramicroporous Polymers for Trace Sulfur Dioxide Capture with High Capacity and Selectivity. Angew. Chem., Int. Ed. 2021, 60 (13), 6986–6991. 10.1002/anie.202013448. 33382169

[ref45] Smith B. The infrared spectroscopy of Alkenes. Spectroscopy 2016, 31 (11), 28–34.

[ref46] Wang Y.; Lan J.; Yang X.; Zhong S.; Yuan L.; Li J.; Peng J.; Chai Z.; Gibson J. K.; Zhai M.; et al. Superhydrophobic Phosphonium Modified Robust 3D Covalent Organic Framework for Preferential Trapping of Charge Dispersed Oxoanionic Pollutants. Adv. Funct. Mater. 2022, 32 (36), 2205222 10.1002/adfm.202205222.

[ref47] Ding M.; Chen L.; Xu Y.; Chen B.; Ding J.; Wu R.; Huang C.; He Y.; Jin Y.; Xia C. Efficient capture of Tc/Re(VII, IV) by a viologen-based organic polymer containing tetraaza macrocycles. Chem. Eng. J. 2020, 380, 122581 10.1016/j.cej.2019.122581.

[ref48] Shen N.; Yang Z.; Liu S.; Dai X.; Xiao C.; Taylor-Pashow K.; Li D.; Yang C.; Li J.; Zhang Y.; et al. ^99^TcO_4_^–^ removal from legacy defense nuclear waste by an alkaline-stable 2D cationic metal organic framework. Nat. Commun. 2020, 11 (1), 5571 10.1038/s41467-020-19374-9. 33149147 PMC7642432

[ref49] Li J.; Chen L.; Shen N.; Xie R.; Sheridan M. V.; Chen X.; Sheng D.; Zhang D.; Chai Z.; Wang S. Rational design of a cationic polymer network towards record high uptake of ^99^TcO_4_^–^ in nuclear waste. Sci. China Chem. 2021, 64 (7), 1251–1260. 10.1007/s11426-020-9962-9.

[ref50] Kang K.; Liu S.; Zhang M.; Li L.; Liu C.; Lei L.; Dai X.; Xu C.; Xiao C. Fast Room-Temperature Synthesis of an Extremely Alkaline-Resistant Cationic Metal-Organic Framework for Sequestering TcO_4_^–^ with Exceptional Selectivity. Adv. Funct. Mater. 2022, 32 (48), 2208148 10.1002/adfm.202208148.

[ref51] Tougerti A.; Cristol S.; Berrier E.; Briois V.; La Fontaine C.; Villain F.; Joly Y. XANES study of rhenium oxide compounds at the L1 and L3 absorption edges. Phys. Rev. B 2012, 85 (12), 125136 10.1103/PhysRevB.85.125136.

[ref52] Mohammad M.; Sherman W. Infrared and Raman spectra of ReO_4_^–^ isolated in alkali halides. J. Phys. C: Solid State Phys. 1981, 14 (3), 283 10.1088/0022-3719/14/3/011.

[ref53] Serre C.; Bourrelly S.; Vimont A.; Ramsahye N. A.; Maurin G.; Llewellyn P. L.; Daturi M.; Filinchuk Y.; Leynaud O.; Barnes P.; et al. An explanation for the very large breathing effect of a metal–organic framework during CO_2_ adsorption. Adv. Mater. 2007, 19 (17), 2246–2251. 10.1002/adma.200602645.

[ref54] Tan K.; Nijem N.; Canepa P.; Gong Q.; Li J.; Thonhauser T.; Chabal Y. J. Stability and hydrolyzation of metal organic frameworks with paddle-wheel SBUs upon hydration. Chem. Mater. 2012, 24 (16), 3153–3167. 10.1021/cm301427w.

[ref55] Wei C.; Yang Z.; Zhang J.; Ji H. Selective and efficient removal of ReO_4_^–^ from aqueous solution by imidazolium-based porous organic polymers. Colloids Surf. A: Physicochem. Eng. 2022, 651, 129754 10.1016/j.colsurfa.2022.129754.

