# Supplementary material for: Creation of Cationic Polymeric Nanotrap Featuring High Anion Density and Exceptional Alkaline Stability for Highly Efficient Pertechnetate Removal from Nuclear Waste Streams
Source: ACS Cent Sci. 2024 Jan 31;10(2):426–38. doi: 10.1021/acscentsci.3c01323 (PMC10906250; doi:10.1021/acscentsci.3c01323)
Supplement: Supplementary file 1 — oc3c01323_si_001.pdf [file oc3c01323_si_001.pdf]

Supporting Information

Creation of Cationic Polymeric Nanotrap Featuring High Anion Density and Exceptional Alkaline Stability for Highly Efficient Pertechnetate Removal from Nuclear Waste Streams

Bin Wang,[†,‡#] Jie Li,[‡,#] Hongliang Huang,[§,#] Bin Liang,[*,†] Yin Zhang,[†] Long Chen,[‡] Kui Tan,[†] Zhifang Chai,[‡] Shuao Wang,[*,‡] Joshua T. Wright,[‖] Robert W. Meulenberg,[⊥] and Shengqian Ma[*,†]

†. Department of Chemistry, University of North Texas 1508W Mulberry St, Denton, TX 76201, U.S.A.

‡. State Key Laboratory of Radiation Medicine and Protection, School for Radiological and Interdisciplinary Sciences (RAD-X), and Collaborative Innovation Center of Radiation Medicine of Jiangsu Higher Education Institutions, Soochow University, Suzhou 215123, China

§. State key laboratory of Separation Membranes and Membrane Processes, Tiangong University, Tianjin 300387, China

|| Department of Physics, Illinois Institute of Technology, Chicago, IL 60616, USA

⊥ Department of Physics and Astronomy and Frontier Institute for Research in Sensor Technologies, University of Maine, Orono, ME 04469, USA

Email: bin.liang@unt.edu; shuaowang@suda.edu.cn; shengqian.ma@unt.edu

Caption of Content

Material characterization 3

Synthesis of monomers 3

Synthesis of CPNs 4

Calculation of the theoretical Cl^- densities 4

Alkaline stability exploration 5

Alkaline and irradiation stability exploration 5

Sorption isotherm investigations 6

Batch experiments 7

Sorption kinetics study 7

Computational methods 8

Supplementary Figures 10

Supplementary Tables 21

References 25

Material characterization

All general chemicals and solvents (AR grade) were commercially available and used as received. FT-IR data were recorded on a SHIMADZU IR Affinity-1 instrument. A Bruker Avance III 400 HD instrument with a magic-angle spinning frequency of 80 kHz was used to collect the solid-state ^{13}C NMR spectroscopy of the polymers. The SEM spectra were obtained by an EVO 18 scanning electron microscope. X-ray photoelectron spectroscopy (XPS) data were recorded on ESCALAB 250Xi with the Al Kα radiation, and energy calibration based on surface contamination C1s (284.8 eV) was applied to analyze the materials with XPS peak. X-ray absorption fine structure (XAFS) experiments were performed at Sector 10 on the insertion device line operated by the Materials Research Collaborative Access Team (MRCAT)[1]. Measurements were performed in transmission on pure materials in Kapton tape. XAFS data processing and analysis were done using the IFEFFIT suite of programs[2]. Initial estimates of the threshold energy values (E_0) were obtained via the inflection point in the normalized absorption edges. A Hanning window was applied to a selected k-range (3-11 $Å^{-1}$) to obtain the Fourier-transformed extended XAFS (EXAFS) data. While the exact structure of the Re is unknown, we use a Re-O path in our fitting model and use FEFF6 to calculate the photoelectron scattering path amplitudes, F_i(k), and phase, ϕ(k), and the sample was fit to the EXAFS equation:

$$x(k) = \frac{N_i S_o^2}{2kR_i^2} F_i(k) e^{-2k^2\sigma_i^2} \sin[2kR_i + \phi_i(k)] \qquad (1)$$

Synthesis of monomers

The ionic monomers utilized in this study, namely 3,3'-(1,4-phenylenebis(methylene))bis(1-vinyl-1H-imidazol-3-ium) (M-1), 3,3'-((5-(1-vinyl-1H-3l4-imidazol-3-yl)-1,3-phenylene)bis(methylene))bis(1-vinyl-1H-imidazol-3-ium) (M-2), and 3,3',3'',3'''-(benzene-1,2,4,5-tetrayltetrakis(methylene))tetrakis(1-vinyl-1H-imidazol-3-ium) (M-3), were synthesized based on previously reported literature with minor modifications

(Supplementary Figure 1-3)[3-4]. To illustrate the synthesis process, we take the example of M-3: 1,2,4,5-tetrakis(bromomethyl)benzene (1.00 g, 2.2 mmol), 1-vinylimidazole (0.92 g, 9.8 mmol), and BHT (0.05 g, 0.2 mmol) were dissolved in CH_3CN (50 mL) in a 250 mL one-necked round-bottom flask. The flask was equipped with a water condenser and heated at 60°C for 48 hours. After cooling the flask to room temperature, the crude product was filtered and washed with CH_3CN (20 mL). Finally, the product was dried under vacuum at 40 ºC for 24 hours. (yield: 1.54 g, 85.0%).

Synthesis of CPNs

The synthesis of the CPNs, namely CPN-1, CPN-2, and CPN-3, was conducted based on a previously published method with slight modifications[3-4]. Taking CPN-3 as an example, M-3 (1.0 g) and AIBN (0.01 g) were dissolved in a mixture of water (10 mL) and DMF (2 mL) in a 20 mL autoclave. The autoclave was heated at 100°C for 24 hours in the oven. After being cooled to room temperature, the sample was washed with water (30 mL) and EtOH (30 mL), followed by fully ions exchanging with NaCl aqueous solution (1 M), the as-prepared sample was vacuum drying at 50°C for 24 hours (yield: approximately 0.71 g, 90.0%).

Calculation of the theoretical Cl^- densities

Given that the three CPNs are produced via free radical polymerization of building units containing -C=C- bonds, with AIBN serving as an initiator in DMF, the structures of these CPNs are composed entirely of a single repeating unit (Figure S4). Consequently, the theoretical Cl^- ion content in these CPNs can be estimated by approximating the Cl^- content in the building unit using Eq 2:

$$N = \frac{m}{Mw_{sk} + Mw_{Cl^-}} \times 1000 \qquad (2)$$

where N (mmol/g) represents the theoretical Cl^- ion content in the CPNs, m represents the amount of Cl^- in the repeating unit, and Mw_{sk} (g/mol) and Mw_{Cl-} (g/mol) represents the molecular weight of the skeleton and the counter Cl^- ions in the repeating unit, respectively.

Alkaline stability exploration

To characterize the alkaline stability of the monomers, we employed ^1H NMR due to the monomers' solubility in water. During the experimental process, we dissolved 20 mg of monomers in 0.5 mL D_2O/1M NaOH D_2O solutions using ultrasonication for 2 minutes in an NMR tube, facilitating the dissolution of the monomers. Subsequently, we performed in-situ ^1H NMR measurements to monitor changes in peak intensities and chemical shifts of the H atoms. To characterize the alkaline stability of the CPNs, we employed FT-IR due to their insolubility in water. In the experimental setup, 20 mg of each POP was immersed in NaOH aqueous solutions with concentrations of 1M, 3M, and 5M, respectively, for a duration of 24 hours. Subsequently, the CPNs were filtered and, prior to drying in an oven (60 °C), washed multiple times with 1M NaCl aqueous solutions. This washing step aimed to exchange the OH^- within the pores of the materials with Cl^- to prevent the effects of OH^- in the FT-IR spectrum.

Alkaline and irradiation stability exploration

FT-IR was utilized to characterize the alkaline and irradiation stability of the CPNs, whereby the well-activated CPN-3 was immersed in DI water or 1 M NaOH aqueous solutions for a duration of 24 hours, and subsequently filtered to obtain the water-treated or NaOH-treated samples. These wet samples together with dry pristine CPN-3 underwent

further treatment under γ-ray irradiation to produce the γ-ray treated samples. Following a drying process in an oven at 60°C for 24 hours, FT-IR spectra were collected for each of the aforementioned samples.

Sorption isotherm investigations

The adsorption isotherms were obtained by mixing 10 mg CPNs with 50 mL ReO_4^- solution of different concentrations from 10 to 300 mg/L at a constant temperature of 298 K with stirring for 12 h. The concentration of ReO_4^- in solution was measured on an inductively coupled plasma-atomic emission spectrometry (ICP-AES, Thermo Fisher Scientific iCAP 7000). The amount of ReO_4^- adsorbed on the POPs was calculated using the mass balance with eq 3:

$$Q_e = \frac{(C_0 - C_e)V}{M} \qquad (3)$$

where Q_e (mg/g) is the equilibrium adsorbed amount; C_0 and C_e (mg/L) are the initial and equilibrium concentrations of adsorbate; V (L) is the volume of solution; and M (g) is the mass of the sorbent. Two isotherm models, including Langmuir isotherm model (4) and Freundlich model (5) were used to fit the sorption data and can be expressed in the following equations:

Langmuir model:

$$\frac{C_e}{Q_e} = \frac{1}{Q_m K_L} + \frac{C_e}{Q_m} \qquad (4)$$

Freundlich model:

$$\ln Q_e = \ln K_F + \frac{1}{n}\ln C_e \qquad (5)$$

where C_e (mg/L) is the equilibrium concentration of adsorbate; Q_e (mg/g) is the equilibrium adsorbed amount; Q_m (mg/g) is the maximum monolayer adsorption capacity; K_L

(L/mg) is the Langmuir constant related to the free energy of adsorption; K_F ($L^n/mol^{n-1}g$) is the Freundlich adsorption constant; and 1/n is a measure of adsorption intensity ranging between 0 and 1.

Batch experiments

Typically, 10 mg of CPNs were mixed with 10 mL of $^{99}TcO_4^-/ReO_4^-$ solution in a glass beaker. The mixtures were stirred at room temperature for 2 h. These mixtures were subsequently separated by a 0.22 μm nylon membrane filter. The concentration of $^{99}TcO_4^-$ in solution was determined by monitoring its activity on a liquid scintillation counting (LSC) system (Perkin Elmer Quantulus 1220). The concentration of ReO_4^- in solution was measured on an inductively coupled plasma-atomic emission spectrometry (ICP-AES, Thermo Fisher Scientific iCAP 7000). The removal efficiency (S%) and distribution coefficient (K_d) of the sorbent were calculated via the following equations:

$$S\% = \frac{C_0 - C_e}{C_0} \times 100\% \qquad (6)$$

$$K_d = \frac{(C_0 - C_e)V}{MC_e} \qquad (7)$$

Where C_0 and C_e (mg/L) are the initial and equilibrium concentrations of adsorbate; V (L) is the volume of solution; and M (g) is the mass of the sorbent.

Sorption kinetics study

Typically, 20 mg of CPNs was mixed with 100 ml of ReO_4^- aqueous solution (28 ppm) in a beaker, the mixture was separated and analyzed after being stirred for a certain time (1 min, 3min, 6 min, 10 min, and 20 min). Besides, the sorption kinetics of CPN-3 at a high solid/liquid ratio (1.0 g/L) was carried out: 20 mg of CPN-3 were mixed 20 ml of $^{99}TcO_4^-$

/ReO_4^- stock solution (28 ppm) in a beaker, the mixture was separated and analyzed after stirred for a certain time (1.5 min, 3 min, 5 min, 10 min, 15 min, 20 min, 30 min, 60 min, 90 min, 120 min, and 180 min). The Pseudo-first-order and Pseudo-second-order model were used to analyze sorption kinetics. The two models are expressed as followed:

Pseudo-first-order model:

$$\ln(Q_e - Q_t) = \ln Q_e - k_1 t \quad (8)$$

Pseudo-second-order model:

$$\frac{t}{Q_t} = \frac{1}{k_2 Q_e^2} + \frac{t}{Q_e} \quad (9)$$

where k_1 (min-1) and k_2 (g/(mg min)) are constants of pseudo-first-order model and pseudo-second-order model, respectively. Q_t and Q_e are the sorption capacity at time t and equilibrium time, respectively.

Computational methods

Density functional theory (DFT) calculations were carried out using the Gaussian 16 program.[5] As for the ring open reaction, geometry optimization calculations were performed using Becke three parameters hybrid exchange-correlation functional (B3LYP) density functional implemented.[6] The 6-31G(d) basis set was employed for C, H, O, and N atoms. The dispersion correction schemes by Grimme (denoted as D3) were used to account for the van der Waals interactions.[7] For the geometry optimization procedure, the structures were optimized until the forces were < 10^{-5} hartree/bohr and the energy change was < 10^{-7} hartree. Based on the optimized reaction and product, single-point energies were then performed at the high precision electronic energy calculated at B3LYP-D3/6-311++G(d,p) level. Zero-point energies, thermal contributions to enthalpies, and Gibbs free energies were derived from vibrational frequency calculations with the same level of theory. Transition structures were

located using the Berny algorithm.[8] The connection between transition structures and minima (its reactants and products) were established by the intrinsic reaction coordinate (IRC) calculations based on the reaction path following algorithm of Gonzalez and Schlegel as coded in Gaussian 16.[9-10] We verified that none of the stationary points have imaginary frequencies and each transition state have only one imaginary frequency.

As for the electrostatic potentials of $^{99}TcO_4^-$ and Fragment-1 were calculated using the DMol3 module of Materials Studio. The B3LYP was used to describe the exchange-correlation interaction. The double numerical plus polarization (DNP) was used to expand electronic wave function. The self-consistent field (SCF) calculations were used and the convergence criterion is 10^{-5} Ha in energy.

As for the adsorption of anion, the Fragment 1 was used as the model for mimicking the positively charged local structure of the amorphous sorbent material. Three anions, including TcO_4^-, SO_4^{2-}, and NO_3^- were used as the sorbates. The isolated structures of each anion and the complex of M^+A^- ($A^- = TcO_4^-$, SO_4^{2-}, or NO_3^-) were fully optimized using B3LYP-D3/6-31G(d)/SDD. The 6-31G(d) basis set was employed for C, H, O, N, and S atoms, and the Stuttgart/Dresden relativistic effective core potentials (SDD) and corresponding valance basis sets for the Tc atom.[11] A high precision single-point energy calculations were performed for these optimized structures in water phase at B3LYP-D3/6-311++G(d,p) level. The SMD implicit solvent model was employed to consider the solvent effect.[12] Based on the single-point energy calculations, the binding energy, ΔE, were calculated by the following equation:

$$\Delta E = E_{complex} - E_{M^+} - E_{anion} \qquad (10)$$

where E_{complex}, $E_{\text{M+}}$, and E_{anion} denote the total energy of each complex, M^+, and anions, respectively.

Supplementary Figures

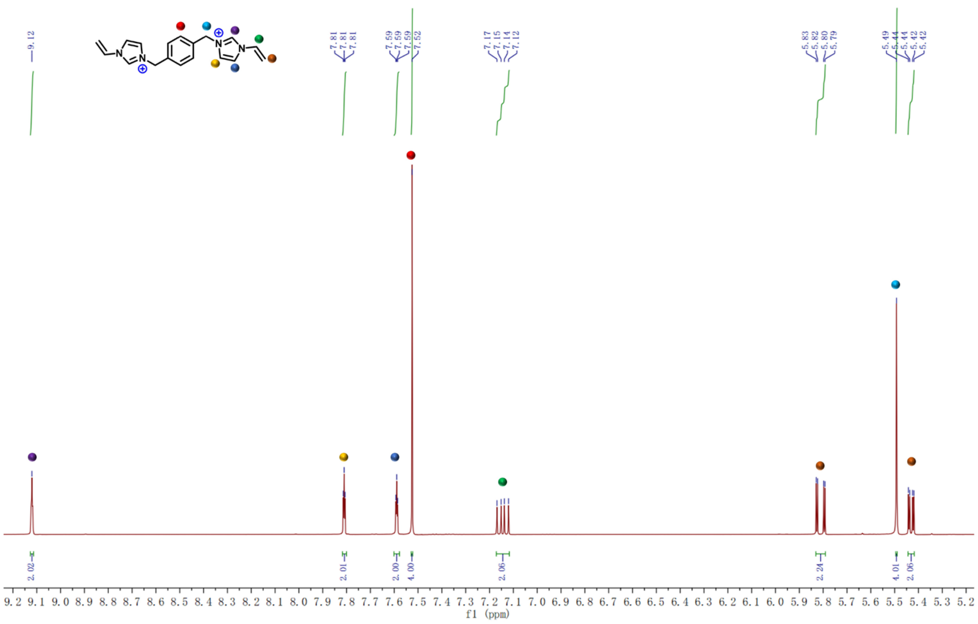

Figure S1. ^{1}H NMR of monomer M-1.

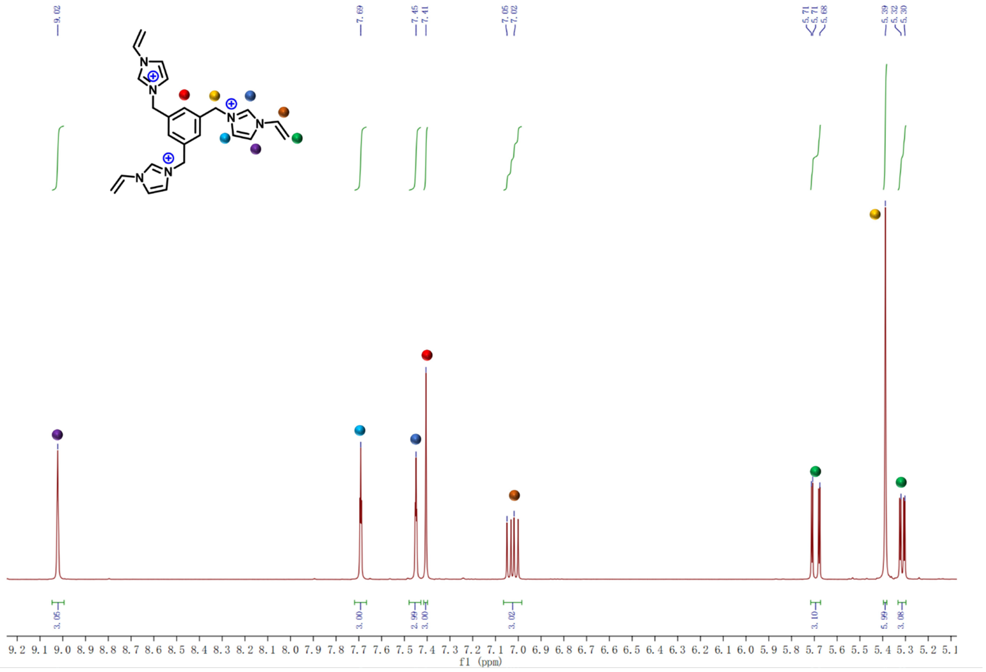

Figure S2. ^{1}H NMR of monomer M-2.

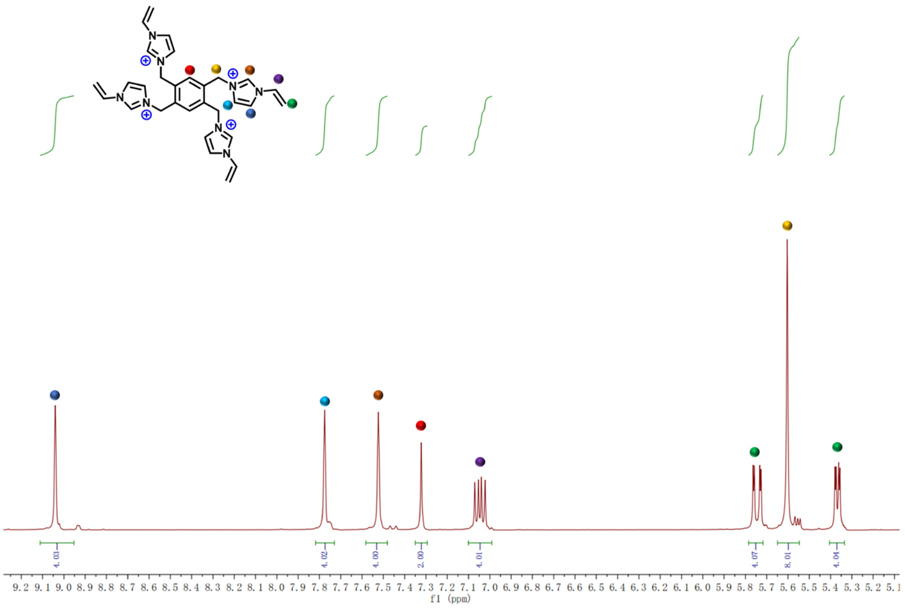

Figure S3. ^{1}H NMR of monomer M-3.

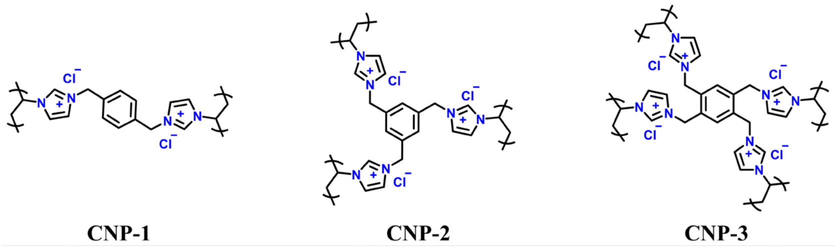

Figure S4. Structure of CPN-1, CPN-2, and CPN-3.

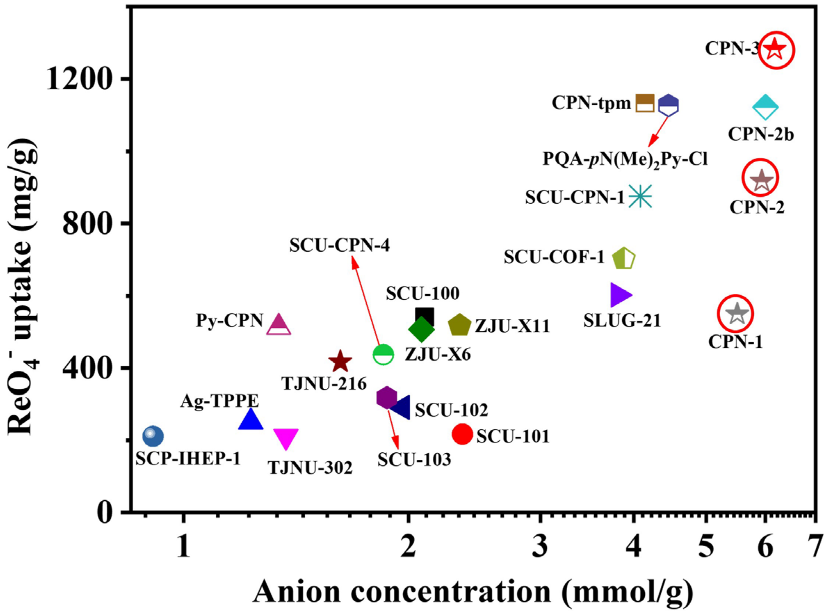

Figure S5. Comparison of the concentration of anions and uptake amount toward ReO_4^- of CPN-1, CPN-2, and CPN-3 with the reported cationic materials.

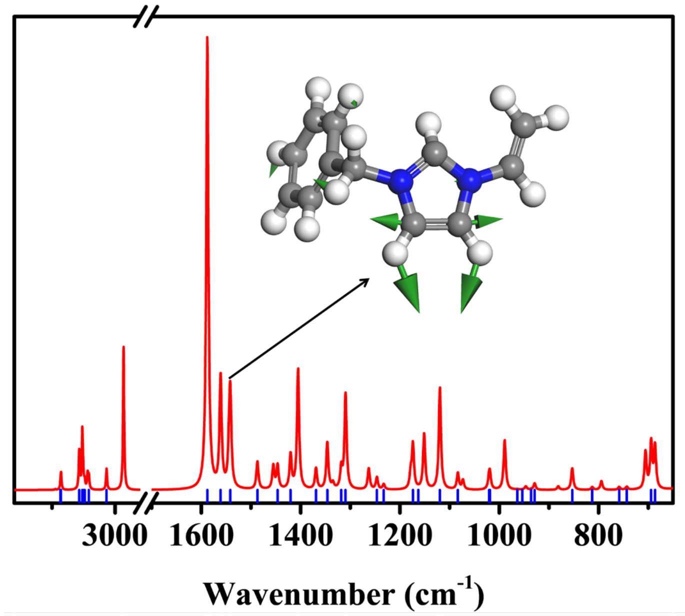

Figure S6. Simulated IR spectrum of typical fragments of the monomers with *ν*(C=C) vibration mode of the azole ring shown.

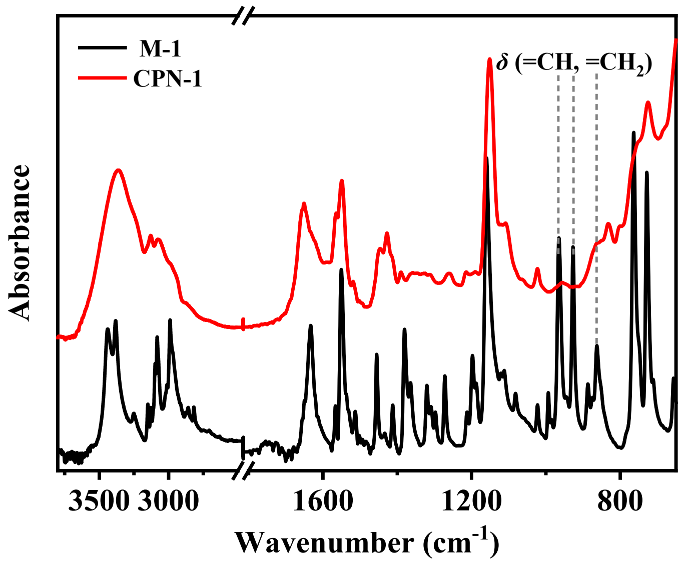

Figure S7. FT-IR spectra of M-1 and CPN-1.

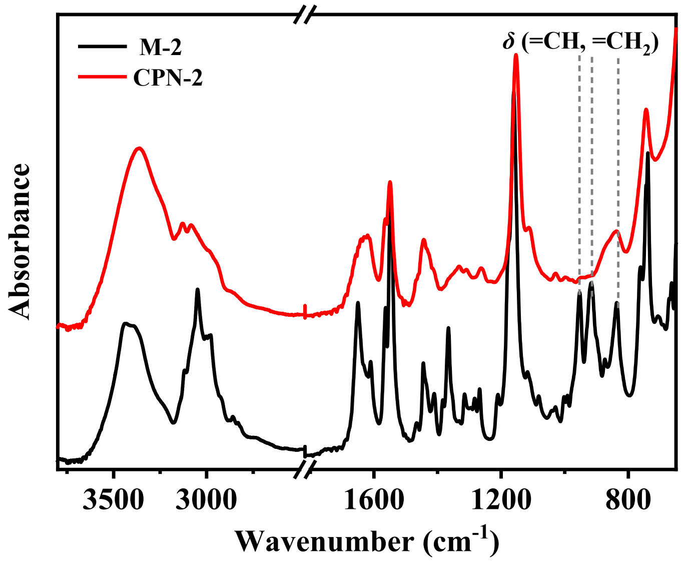

Figure S8. FT-IR spectra of M-2 and CPN-2.

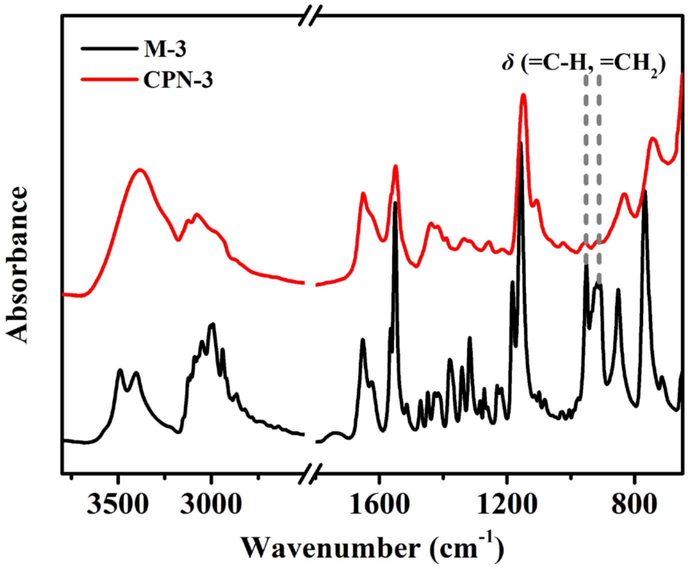

Figure S9. FT-IR spectra of M-3 and CPN-3.

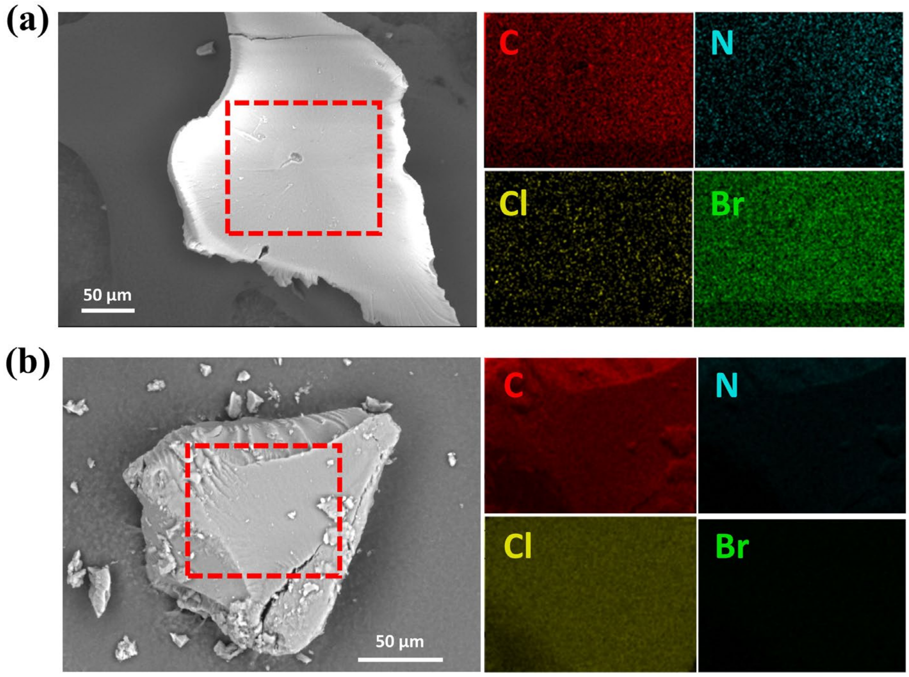

Figure S10. SEM image and EDS mapping of (a) Br-CPN-1 and (b) CPN-1.

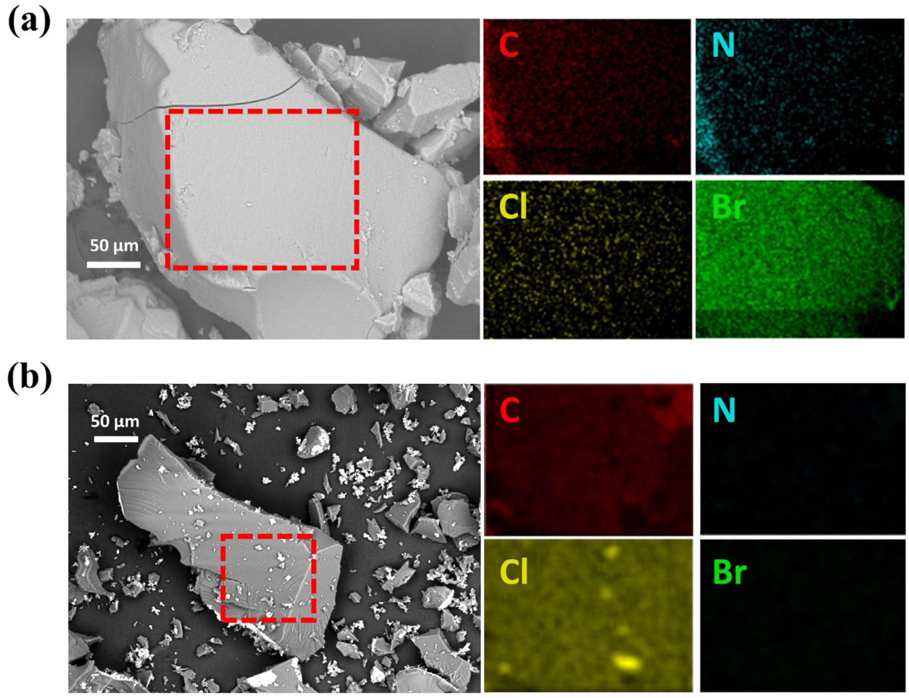

Figure S11. SEM image and EDS mapping of (a) Br-CPN-2 and (b) CPN-2.

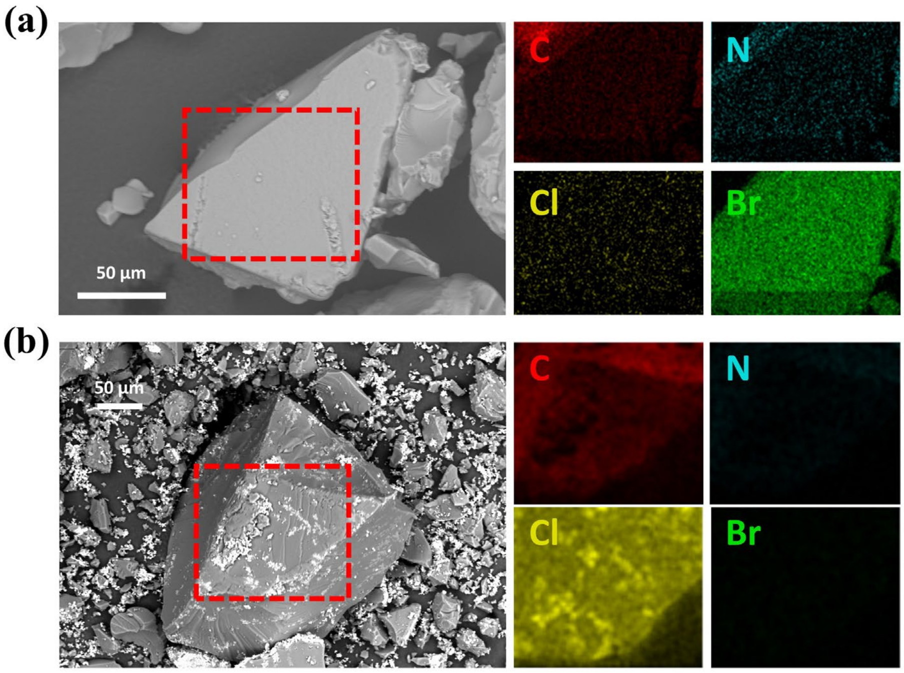

Figure S12. SEM image and EDS mapping of (a) Br-CPN-3 and (b) CPN-3.

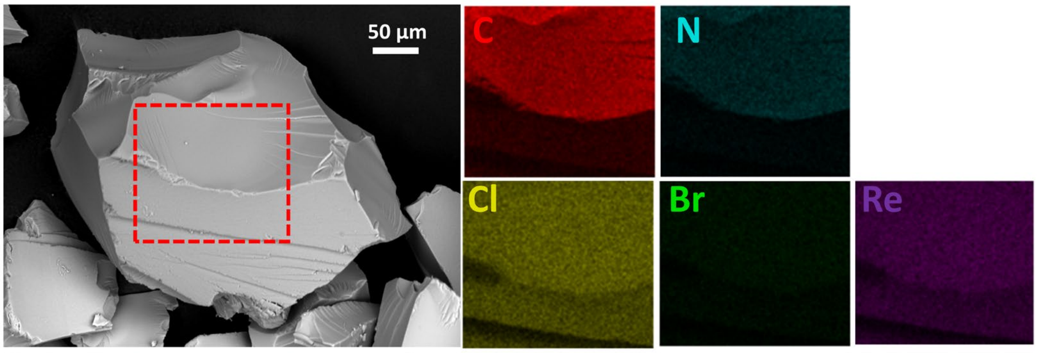

Figure S13. SEM image and EDS mapping of CPN-3@ReO_4^-.

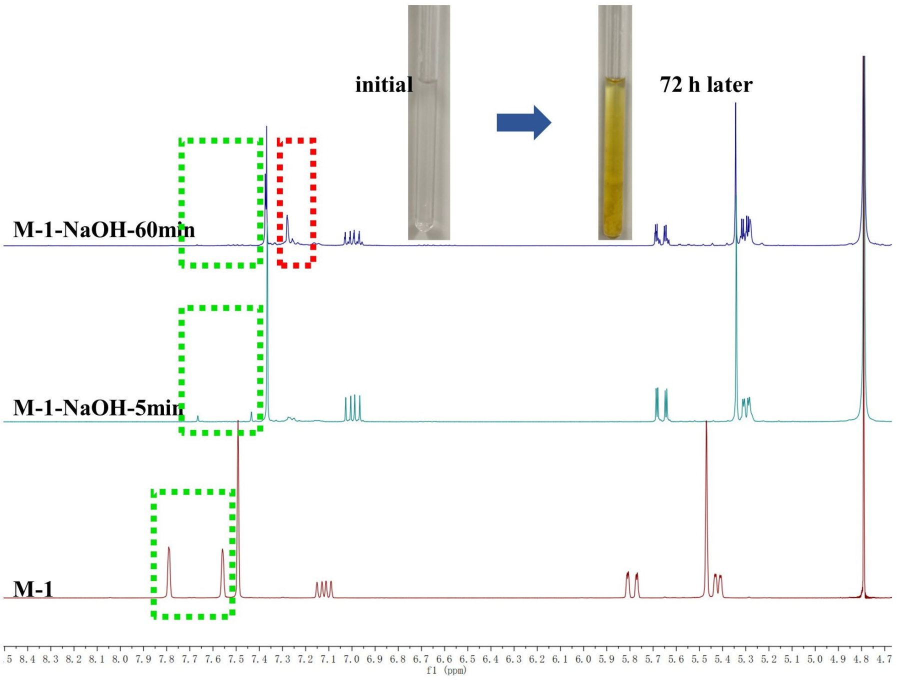

Figure S14. Changes of ^{1}H NMR spectrum of M-1 immersed in 1M NaOH D_2O solution along with time.

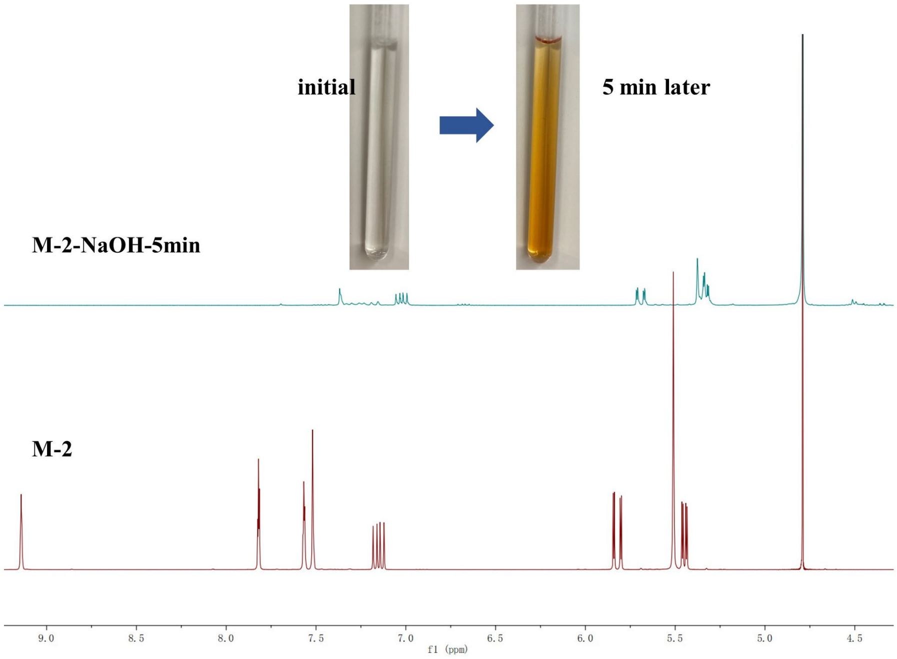

Figure S15. Changes of ^{1}H NMR spectrum of M-2 immersed in 1M NaOH D_2O solution along with time.

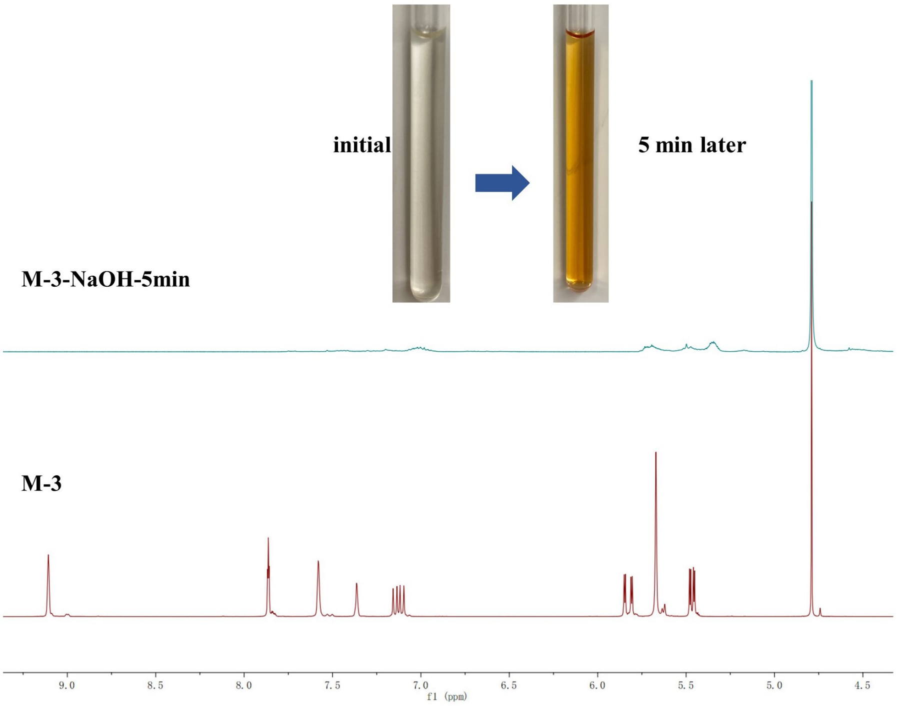

Figure S16. Changes of ^{1}H NMR spectrum of M-3 immersed in 1M NaOH D_2O solution along with time.

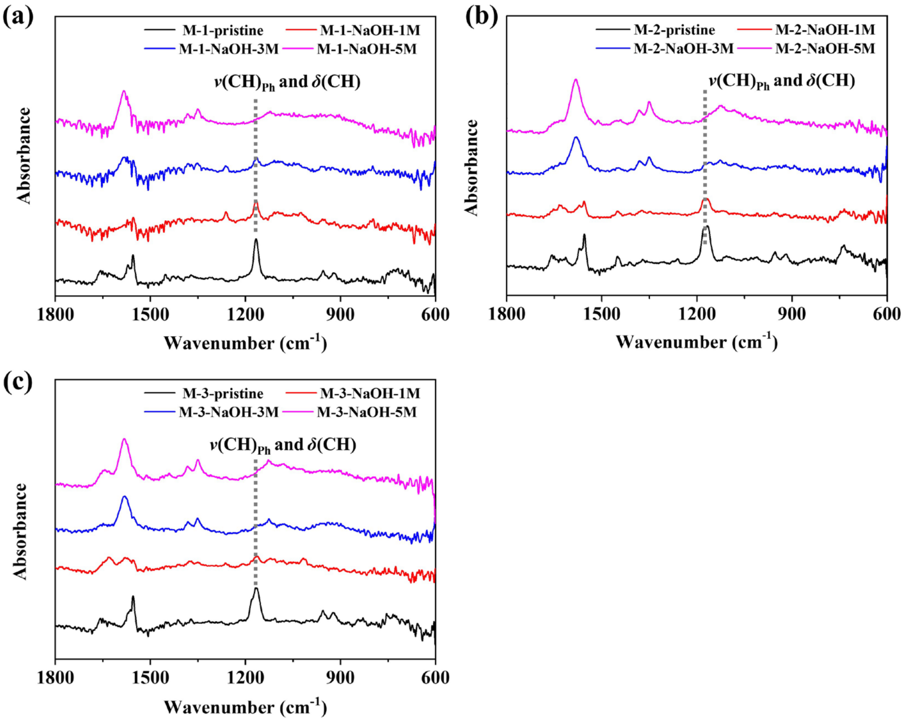

Figure S17. FT-IR spectra of (a) M-1 (b) M-2, and (c) M-3 after being treated by 1 M, 3 M, and 5 M NaOH aqueous solutions for 60 min.

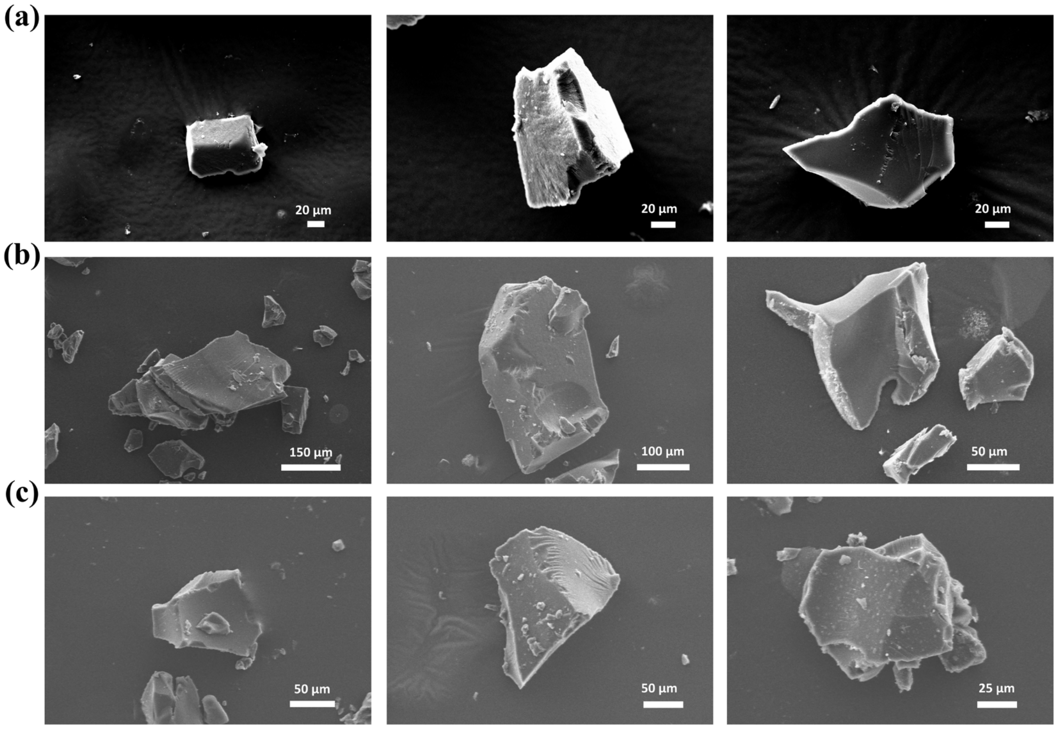

Figure S18 SEM images of (a) CPN-1, (b) CPN-2, and (c) CPN-3 after being treated with 1 M (left), 3 M (middle), and 5 M (right) NaOH aqueous solutions for 24 hours.

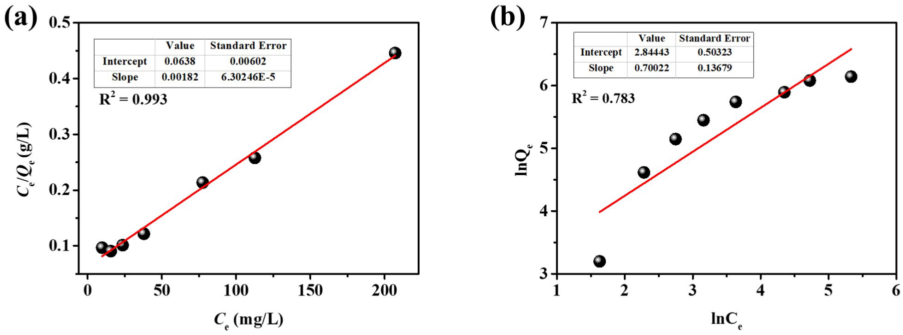

Figure S19. Sorption isotherm fitting for ReO_4^- of CPN-1. (a) Langmuir model and (b) Freundlich model.

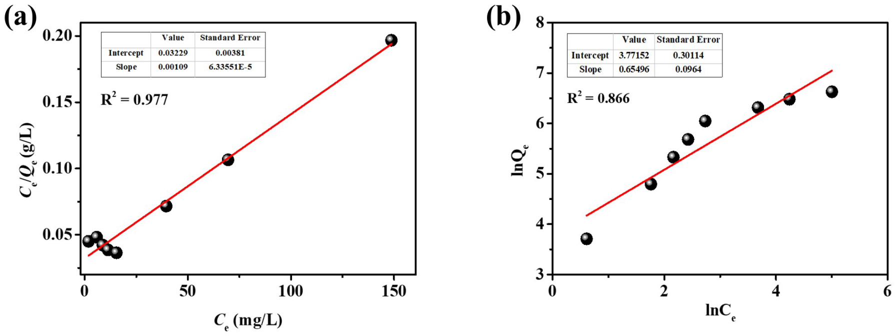

Figure S20. Sorption isotherm fitting for ReO_4^- of CPN-2. (a) Langmuir model and (b) Freundlich model.

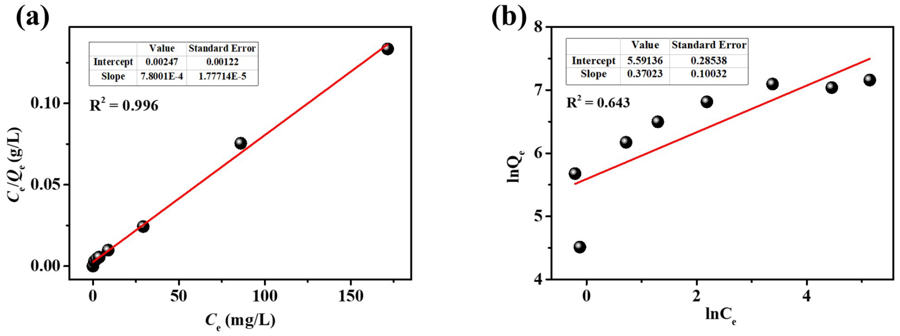

Figure S21. Sorption isotherm fitting for ReO_4^- of CPN-3. (a) Langmuir model and (b) Freundlich model.

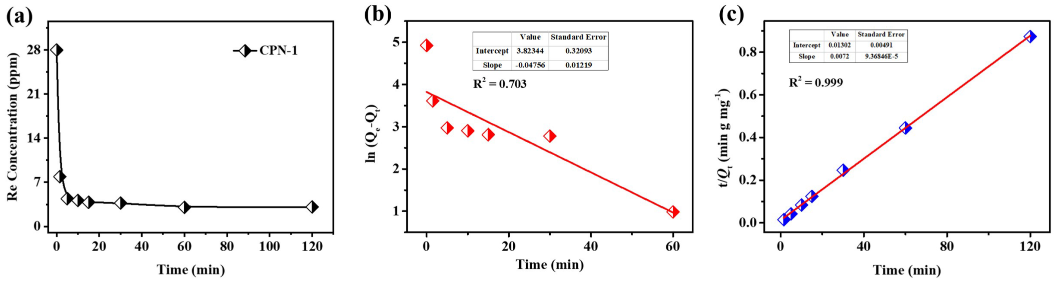

Figure S22. Sorption kinetics and fitting of ReO_4^- by CPN-1. (a) Sorption kinetics of ReO_4^- by CPN-1. Conditions: $[Re]_{initial}$ = 28 ppm and $m_{sorbent}/V_{solution}$ = 0.2 g/L. (b) Pseudo-first-order model and (c) Pseudo-second-order model for ReO_4^- by CPN-1.

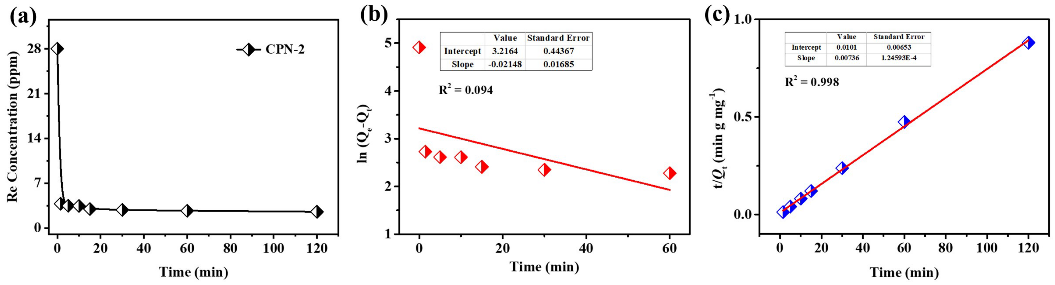

Figure S23. Sorption kinetics and fitting of ReO_4^- by CPN-2. (a) Sorption kinetics of ReO_4^- by CPN-2. Conditions: $[Re]_{initial}$ = 28 ppm and $m_{sorbent}/V_{solution}$ = 0.2 g/L. (b) Pseudo-first-order model and (c) Pseudo-second-order model for ReO_4^- by CPN-2.

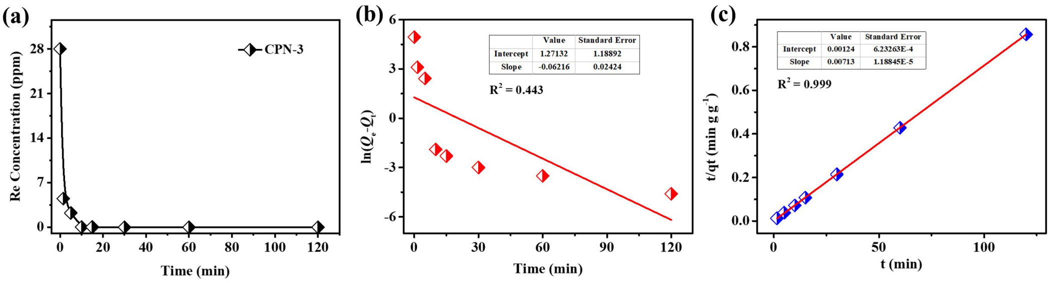

Figure S24. Sorption kinetics and fitting of ReO_4^- by CPN-3. (a) Sorption kinetics of ReO_4^- by CPN-3. Conditions: $[Re]_{initial}$ = 28 ppm and $m_{sorbent}/V_{solution}$ = 0.2 g/L. (b) Pseudo-first-order model and (c) Pseudo-second-order model for ReO_4^- by CPN-3.

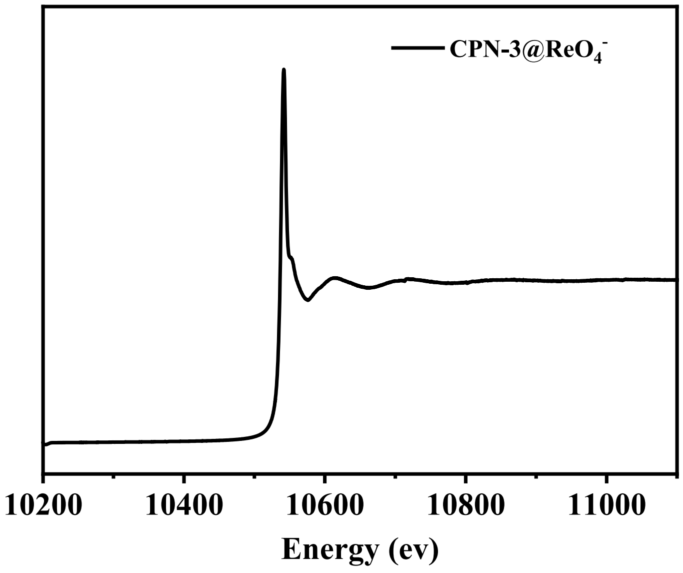

Figure S25. Re L_3–edge XANES spectra for CPN-3@ReO_4^-

Supplementary Tables

Table S1. Comparison of the concentration of anions and uptake amount toward ReO_4^- of the reported cationic materials.

Sorbent	Type of anion	Concentration (mmol/g)	Uptake amoung (mg/g)	Ref.
SCU-100	NO_3^-	2.10	541	13
SCU-101	NO_3^-	2.36	217	14
Ag-TPPE	NO_3^-	1.23	251	15
TJNU-302	SbF_6^-	1.37	211	16
ZJU-X6	NO_3^-	2.08	507	17
SCU-102	NO_3^-	1.96	291	18
SLUG-21	ethanedisulfonate	3.81	602	19
SCU-103	NO_3^-	1.87	318	20
TJNU-216	$CF_3SO_3^-$	1.62	417	21
ZJU-X11	NO_3^-	2.34	518	22
SCP-IHEP-1	NO_3^-	0.91	211	23
SCU-CPN-1	Cl^-	4.08	876	24
CPN-tpm	Cl^-	4.14	1133	25
SCU-CPN-4	Cl^-	1.85	437	26
Py-CPN	Cl^-	1.34	513	27
CPN-2b	Cl^-	6.00	1122	28
PQA-*p*N(Me)$_2$Py-Cl	Cl^-	4.45	1127	29

SCU-COF-1	Cl^-	3.88	702	30
CPN-1	Cl^-	5.50	549	This work
CPN-2	Cl^-	5.93	917	
CPN-3	Cl^-	6.17	1282	

Table S2. Fitting results based on the Langmuir and Freundlich models.

Sample	Langmuir			Freundlich		
	Q_m (mg/g)	K_L (L/mg)	R^2	K_F ($L^n/mol^{n-1}g$)	n	R^2
CPN-1	549	0.028	0.993	17.19	1.43	0.783
CPN-2	917	0.034	0.977	43.45	1.53	0.866
CPN-3	1282	0.316	0.996	268.09	2.70	0.643

Table S3. Fitting results based on Pseudo-first-order and Pseudo-second-order model

Sample	Pseudo-first-order			Pseudo-second-order		
	k_1 (min^{-1})	q_e (mg/g)	R^2	k_2 (g mg^{-1} min^{-1})	q_e (mg/g)	R^2
CPN-1	0.048	45.761	0.703	3.98×10^{-3}	138.8	0.999
CPN-2	0.022	24.938	0.094	5.36×10^{-3}	135.87	0.998
CPN-3	0.062	3.566	0.443	4.10×10^{-2}	140.25	0.999

Table S4. Comparison of the experimental conditions, equilibrium time, rate constant, distribution coefficient, and sorption capacity of CPN-3 with other TcO_4^-/ReO_4^- sorbents.

Type	Sorbents	Experimental conditions			Equilibrium time (min)	Kintics k_2 (g mg^{-1} min^{-1})	Distribution coefficient (mL/g)	Sorption capacity (mg/g)	Rf.
		C_0 (mg/L)	pH	Solid/liquid ratio (g/L)					
Inorganic materials	NDTB-1	1.455× 10^{-4} M	7	3.3	36 h	0.059 s^{-1} M^{-1}	1.05 × 10^4	162.2	31-32
	ASBC	20	1	3.0	480	8.30 × 10^{-3}	0.57	14.6	33
	ZrO_2@rGO	10	4	0.1	24 h	0.012 g/(mg·h)	-	43.5	34
	GO-DEA-DIBA	20	2	2.0 g/L	24 h	4.79 × 10^{-3}	-	141	35
Resin	Purolite A530E	28	7	1.0	150	6.75 × 10^{-3}	-	707	36
	Purolite A532E	28	7	1.0	150	4.60 × 10^{-3}	-	446	
	R_2SO_4 resin	200	6	4	60	0.0165	-	354	37
	Resin D318	320	5	1.0	115	6.37×10^{-4} s^{-1}	-	351	38
Metal-Orgniac Frameworks	SBN	28	7	0.5	10	-	-	786	39
	SCU-100	28	7	1.0	30	-	1.9 × 10^5	541	13
	SCU-101	28	7	1.0	10	-	7.5 × 10^5	217	14
	Ag-TPPE	20	7	1.0	40	0.0186	3.93 × 10^5	251	15
	NCU-3-Br	20	7	1.0 g/L	10	0.061 min^{-1}	-	483	40
	NU-1000	27	7	0.3	5	-	-	210	41
	TJNU-302	1 mol/L	7	3.7	10	0.1022	5.8 × 10^5	211	16

	ZJU-X6	30	7	1.0	20	-	-	507	17
	SCU-102	28	7	1.0	20	2.48×10^{-2}	5.6×10^{5}	291	18
	SLUG-21	1.8×10^{3}	7	1.6	48 h	-	-	602	19
	SCU-103	14	7	1.0	5	-	3.47×10^{5}	318	20
	bis-$PC_2(Cl)$@MIL-101	26	7	1.0	0.5	22.03	3.3×10^{6}	362	42
	TJNU-216	72	7	1.5	24 h	2.3×10^{-4}	1.0×10^{5}	417	21
	ZJU-X11	30	7	1.0	10	-	-	518	22
	SCP-IHEP-1	44.6	7	0.5	10	-	2.6×10^{5}	211	23
	UiO-66-NH_3^+	molar ratio of ReO_4^-/PAF-1-F = 1:2			24 h	-	-	159	43
	SCU-CPN-1	28	7	1.0	30 s	-	6.2×10^{5}	876	24
Catonic	SCU-CPN-1	28	7	0.05	10	2.01×10^{-2}			
Polymetic	PAF-1-F	molar ratio of ReO_4^-/PAF-1-F = 1:2			24 h	-	2.55×10^{4}	420	44
Nanotraps	PS-g-4VP-IE	2000	7	20	30	0.018	-	252	45
	CPN-tpm	35	7	0.06	20	3.3×10^{-3}	8.5×10^{5}	1133	25
	SCU-CPN-4	28	7	1.0	1	5.60×10^{-3}	1.5×10^{7}	437	26
	Py-CPN	28	7	0.5	1	0.98	6.65×10^{7}	513	27
	PQA-*p*$N(Me)_2$Py-Cl	50	7	0.04	60	-	1.0×10^{7}	1127	29
	TbDa-COF	500	7	0.5	30	1.05×10^{-2}	-	952	46
	PS-COF-1	56	6	0.05	180	7.36×10^{-5}	-	1262	47

TFAM-BDNP	28	7	0.5	1	-	-	998	48
3DCOF-g-VBPPh$_3$Cl	200	7	0.5	5	2.95×10^{-2}	1.0×10^{8}	181	49
DhaTG$_{Cl}$	25	7	1.0	30	0.43	5.0×10^{5}	437	50
SCU-COF-1	28	7	1.0	1	-	3.89×10^{5}	702	30
VBCOP	45	7	0.25	5	0.05	4.0×10^{5}	444	51
CPN-1	28	7	1.0	1	3.98×10^{-3}	2.8×10^{5}	549	This work
CPN-2	28	7	1.0	1	5.36×10^{-3}	1.8×10^{5}	917	
CPN-3	28	7	1.0	1	4.10×10^{-2}	7.0×10^{7}	1282	

Table S5. Composition of Hanford Low Activity Waste (LAW) Melter Recycle Stream

Anions	Concentration (mol/L)	Molar ratio (Anion: TcO_4^-)
TcO_4^-	1.94×10^{-4}	1.0
NO_3^-	6.07×10^{-2}	314
Cl^-	6.39×10^{-2}	330
NO_2^-	1.69×10^{-1}	873
SO_4^{2-}	6.64×10^{-5}	0.0343
CO_3^{2-}	4.30×10^{-5}	0.222

Table S6. Composition of Savannah River Site (SRS) High-Level Waste (HLW) Stream

Anions	Concentration (mol/L)	Molar ratio (Anion: TcO_4^-)
ReO_4^-/TcO_4^-	7.92×10^{-5}	1.0
NO_3^-	2.6	32819
OH^-	1.33	16788
NO_2^-	1.34×10^{-1}	1691
SO_4^{2-}	6.64×10^{-5}	6576
CO_3^{2-}	4.30×10^{-5}	328

Table S7. Composition of the removal efficiency of CPN-3 toward TcO4- from simulated Hanford LAW and SRS HLW Stream.

Sorbent	TcO_4^- removal from Hanford LAW Stream (%)	TcO_4^-Removal from SRS HLW Stream (%)	Ref.
SCU-CPN-4	97.4 (5g/L)	94.3 (20 g/L)	26
NDTB-1	13.0 (5 g/L)	-	32
	44.8 (25 g/L)		
Ag-TPPE	-	37.0 (1g/L)	15
		90.0 (10g/L)	
SCU-102	95.4 (10 g/L)	-	18
SCU-COF-1	20.9 (1 g/L)	-	30
	62.8 (10 g/L)		
SCU-100	59.3 (1 g/L)	-	13
	87.0 (10 g/L)		
SCU-101	75.2 (10 g/L)	-	14
SCU-103	-	52.0 (5 g/L)	20
		90.0 (40 g/L)	
SCP-IHEP-1	79.2 (0.5 g/L)	-	23
SCU-CPN-1	90.0 (5 g/L)	-	24
SCU-CPN-2	67.0 (5 g/L)	-	28
PS-COF-1	75.3 (5 g/L)	-	47
CPN-3	72.0 (5 g/L)	68.0 (40 g/L)	This work
	84.0 (10 g/L)		

Table S8. EXAFS results of Re (VII) adsorption by SCPN-4@ReO_4^- at Re L_3-edge, T=298 K. C.N. means coordination number of the neighbors, R is the bond distance, and σ^2 is the Debye-Waller factor.

Sample	shell	C.N.	R (Å)	σ^2 (Å^2)
SCPN-4	Re-O	4	1.94 ± 0.01	0.003 ± 0.001

References

(1) Segre, C.; Leyarovska, N.; Chapman, L.; Lavender, W.; Plag, P.; King, A.; Kropf, A.; Bunker, B.; Kemner, K.; Dutta, P., The MRCAT insertion device beamline at the Advanced Photon Source. *AIP Conf. Proc.* **2000,** *521* (1), 419-422.

(2) Ravel, B.; Newville, M., ATHENA, ARTEMIS, HEPHAESTUS: data analysis for X-ray absorption spectroscopy using IFEFFIT. *J. Synchrotron Radiat.* **2005,** *12* (Pt 4), 537-41.

(3) Suo, X.; Cui, X.; Yang, L.; Xu, N.; Huang, Y.; He, Y.; Dai, S.; Xing, H., Synthesis of Ionic Ultramicroporous Polymers for Selective Separation of Acetylene from Ethylene. *Adv. Mater.* **2020,** *32* (29), e1907601.

(4) Suo, X.; Yu, Y.; Qian, S.; Zhou, L.; Cui, X.; Xing, H., Tailoring the Pore Size and Chemistry of Ionic Ultramicroporous Polymers for Trace Sulfur Dioxide Capture with High Capacity and Selectivity. *Angew. Chem. Int. Ed.* **2021,** *60* (13), 6986-6991.

(5) Frisch, M. J.; Trucks, G. W.; Schlegel, H. B.; Scuseria, G. E.; Robb, M. A.; Cheeseman, J. R.; Scalmani, G.; Barone, V.; Mennucci, B.; Petersson, G. A.; Nakatsuji, H.; Caricato, M.; Li, X.; Hratchian, H. P.; Izmaylov, A. F.; Bloino, J.; Zheng, G.; Sonnenberg, J. L.; Hada, M.; Ehara, M.; Toyota, K.; Fukuda, R.; Hasegawa, J.; Ishida, M.; Nakajima, T.; Honda, Y.; Kitao, O.; Nakai, H.; Vreven, T.; Montgomery, J. J. A.; Peralta, J. E.; Ogliaro, F.; Bearpark, M.; Heyd, J. J.; Brothers, E.; Kudin, K. N.; Staroverov, V. N.; Kobayashi, R.; Normand, J.; Raghavachari, K.; Rendell, A.; Burant, J. C.; Iyengar, S. S.; Tomasi, J.; Cossi, M.; Rega, N.; Millam, N. J.; Klene, M.; Knox, J. E.; Cross, J. B.; Bakken, V.; Adamo, C.; Jaramillo, J.; Gomperts, R.; Stratmann, R. E.; Yazyev, O.; Austin, A. J.; Cammi, R.; Pomelli, C.; Ochterski, J. W.; Martin, R. L.; Morokuma, K.; Zakrzewski, V. G.; Voth, G. A.; Salvador, P.; Dannenberg, J. J.; Dapprich, S.; Daniels, A. D.; Farkas, O.; Foresman, J. B.; Ortiz, J. V.; Cioslowski, J.; Fox, D. J., *Gaussian 09, Revision A.02; Gaussian, Inc.: Wallingford CT, 2009.*

(6) Stephens, P. J.; Devlin, F. J.; Chabalowski, C. F.; Frisch, M. J., Ab Initio Calculation of Vibrational Absorption and Circular Dichroism Spectra Using Density Functional Force Fields. *J. Phys. Chem.* **1994,** *98*, 11623-11627.

(7) Grimme, S.; Antony, J.; Ehrlich, S.; Krieg, H., A Consistent and Accurate *ab initio* Parametrization of Density Functional Dispersion Correction (DFT-D) for the 94 Elements H-Pu. *J. Chem. Phys.* **2010,** *132*, 154104.

(8) Schlegel, H. B., Optimization of Equilibrium Geometries and Transition Structures. *J. Comput. Chem.* **1982,** *3*, 214-218.

(9) Fukui, K., A Formulation of the Reaction Coordinate. *J. Phys. Chem.* **1970,** *74*, 4161-4163.

(10) Fukui, K., The Path of Chemical Reactions-The IRC Approach. *Acc. Chem. Res.* **1981,** *14*, 363-368.

(11) Dolg, M.; Wedig, U.; Stoll, H.; Preuss, H., Energy‐adjusted *ab initio* Pseudopotentials for the First Row Transition Elements. *J. Chem. Phys.* **1987,** *86* (2), 866-872.

(12) Marenich, A. V.; Cramer, C. J.; Truhlar, D. G., Universal Solvation Model Based on Solute Electron Density and on a Continuum Model of the Solvent Defined by the Bulk Dielectric Constant and Atomic Surface Tensions. *J. Phys. Chem. B* **2009,** *113*, 6378-6396.

(13) Sheng, D.; Zhu, L.; Xu, C.; Xiao, C.; Wang, Y.; Wang, Y.; Chen, L.; Diwu, J.; Chen, J.; Chai, Z.; Albrecht-Schmitt, T. E.; Wang, S., Efficient and Selective Uptake of TcO_4^- by a Cationic Metal-Organic Framework Material with Open Ag^+ Sites. *Environ. Sci. Technol.* **2017,** *51* (6), 3471-

3479.

(14) Zhu, L.; Sheng, D.; Xu, C.; Dai, X.; Silver, M. A.; Li, J.; Li, P.; Wang, Y.; Wang, Y.; Chen, L.; Xiao, C.; Chen, J.; Zhou, R.; Zhang, C.; Farha, O. K.; Chai, Z.; Albrecht-Schmitt, T. E.; Wang, S., Identifying the Recognition Site for Selective Trapping of $^{99}TcO_4^-$ in a Hydrolytically Stable and Radiation Resistant Cationic Metal-Organic Framework. *J. Am. Chem. Soc.* **2017,** *139* (42), 14873-14876.

(15) Kang, K.; Liu, S.; Zhang, M.; Li, L.; Liu, C.; Lei, L.; Dai, X.; Xu, C.; Xiao, C., Fast Room-Temperature Synthesis of an Extremely Alkaline-Resistant Cationic Metal-Organic Framework for Sequestering TcO_4^- with Exceptional Selectivity. *Adv. Funct. Mater.* **2022,** *32* (48), 2208148.

(16) Li, C. P.; Zhou, H.; Chen, J.; Wang, J. J.; Du, M.; Zhou, W., A Highly Efficient Coordination Polymer for Selective Trapping and Sensing of Perrhenate/Pertechnetate. *ACS Appl. Mater. Inter.* **2020,** *12* (13), 15246-15254.

(17)Kang, K.; Shen, N.; Wang, Y.; Li, L.; Zhang, M.; Zhang, X.; Lei, L.; Miao, X.; Wang, S.; Xiao, C., Efficient sequestration of radioactive 99TcO4- by a rare 3-fold interlocking cationic metal-organic framework: A combined batch experiments, pair distribution function, and crystallographic investigation. *Chem. Eng. J.* **2022,** *427*, 130942.

(18) Sheng, D.; Zhu, L.; Dai, X.; Xu, C.; Li, P.; Pearce, C. I.; Xiao, C.; Chen, J.; Zhou, R.; Duan, T.; Farha, O. K.; Chai, Z.; Wang, S., Successful Decontamination of $^{99}TcO_4^-$ in Groundwater at Legacy Nuclear Sites by a Cationic Metal-Organic Framework with Hydrophobic Pockets. *Angew. Chem. Int. Ed.* **2019,** *58* (15), 4968-4972.

(19) Fei, H.; Bresler, M. R.; Oliver, S. R., A new paradigm for anion trapping in high capacity and selectivity: crystal-to-crystal transformation of cationic materials. *J. Am. Chem. Soc.* **2011,** *133* (29), 11110-11113.

(20) Shen, N.; Yang, Z.; Liu, S.; Dai, X.; Xiao, C.; Taylor-Pashow, K.; Li, D.; Yang, C.; Li, J.; Zhang, Y.; Zhang, M.; Zhou, R.; Chai, Z.; Wang, S., $^{99}TcO_4^-$ removal from legacy defense nuclear waste by an alkaline-stable 2D cationic metal organic framework. *Nat. Commun.* **2020,** *11* (1), 5571.

(21) Li, C.-P.; Ai, J.-Y.; Zhou, H.; Chen, Q.; Yang, Y.; He, H.; Du, M., Ultra-highly selective trapping of perrhenate/pertechnetate by a flexible cationic coordination framework. *Chem. Commun.* **2019,** *55* (12), 1841-1844.

(22) Kang, K.; Li, L.; Zhang, M.; Zhang, X.; Lei, L.; Xiao, C., Constructing Cationic Metal-Organic Framework Materials Based on Pyrimidyl as a Functional Group for Perrhenate/Pertechnetate Sorption. *Inorg. Chem.* **2021,** *60* (21), 16420-16428.

(23) Mei, L.; Li, F. Z.; Lan, J. H.; Wang, C. Z.; Xu, C.; Deng, H.; Wu, Q. Y.; Hu, K. Q.; Wang, L.; Chai, Z. F.; Chen, J.; Gibson, J. K.; Shi, W. Q., Anion-adaptive crystalline cationic material for $^{99}TcO_4^-$ trapping. *Nat. Commun.* **2019,** *10* (1), 1532.

(24) Li, J.; Dai, X.; Zhu, L.; Xu, C.; Zhang, D.; Silver, M. A.; Li, P.; Chen, L.; Li, Y.; Zuo, D.; Zhang, H.; Xiao, C.; Chen, J.; Diwu, J.; Farha, O. K.; Albrecht-Schmitt, T. E.; Chai, Z.; Wang, S., $^{99}TcO_4^-$ remediation by a cationic polymeric network. *Nat. Commun.* **2018,** *9* (1), 3007.

(25) Li, X.; Li, Y.; Wang, H.; Niu, Z.; He, Y.; Jin, L.; Wu, M.; Wang, H.; Chai, L.; Al-Enizi, A. M.; Nafady, A.; Shaikh, S. F.; Ma, S., 3D Cationic Polymeric Network Nanotrap for Efficient Collection of Perrhenate Anion from Wastewater. *Small* **2021,** *17* (20), e2007994.

(26) Li, J.; Li, B.; Shen, N.; Chen, L.; Guo, Q.; Chen, L.; He, L.; Dai, X.; Chai, Z.; Wang, S., Task-Specific Tailored Cationic Polymeric Network with High Base-Resistance for Unprecedented $^{99}TcO_4^-$ Cleanup from Alkaline Nuclear Waste. *ACS Cent. Sci.* **2021,** *7* (8), 1441-1450.

(27) Yan, R.-H.; Cui, W.-R.; Jiang, W.; Huang, J.; Liang, R.-P.; Qiu, J.-D., Rationally designed pyridinium cationic polymeric network for effective TcO_4^-/ReO_4^- remediation. *Chem. Eng. Sci.* **2023,** *268*, 118403.

(28) Li, J.; Chen, L.; Shen, N.; Xie, R.; Sheridan, M. V.; Chen, X.; Sheng, D.; Zhang, D.; Chai, Z.; Wang, S., Rational design of a cationic polymer network towards record high uptake of $^{99}TcO_4^-$ in nuclear waste. *Sci. China Chem.* **2021,** *64* (7), 1251-1260.

(29) Sun, Q.; Zhu, L.; Aguila, B.; Thallapally, P. K.; Xu, C.; Chen, J.; Wang, S.; Rogers, D.; Ma, S., Optimizing radionuclide sequestration in anion nanotraps with record pertechnetate sorption. *Nat. Commun.* **2019,** *10* (1), 1646.

(30) He, L.; Liu, S.; Chen, L.; Dai, X.; Li, J.; Zhang, M.; Ma, F.; Zhang, C.; Yang, Z.; Zhou, R.; Chai, Z.; Wang, S., Mechanism unravelling for ultrafast and selective $^{99}TcO_4^-$ uptake by a radiation-resistant cationic covalent organic framework: a combined radiological experiment and molecular dynamics simulation study. *Chem. Sci.* **2019,** *10* (15), 4293-4305.

(31) Wang, S.; Alekseev, E. V.; Diwu, J.; Casey, W. H.; Phillips, B. L.; Depmeier, W.; Albrecht-Schmitt, T. E., NDTB-1: a supertetrahedral cationic framework that removes TcO_4^- from solution. *Angew. Chem. Int. Ed.* **2010,** *49* (6), 1057-1060.

(32) Wang, S.; Yu, P.; Purse, B. A.; Orta, M. J.; Diwu, J.; Casey, W. H.; Phillips, B. L.; Alekseev, E. V.; Depmeier, W.; Hobbs, D. T.; Albrecht-Schmitt, T. E., Selectivity, Kinetics, and Efficiency of Reversible Anion Exchange with TcO_4^- in a Supertetrahedral Cationic Framework. *Adv. Funct. Mater.* **2012,** *22* (11), 2241-2250.

(33) Hu, H.; Jiang, B.; Zhang, J.; Chen, X., Adsorption of perrhenate ion by bio-char produced from Acidosasa edulis shoot shell in aqueous solution. *RSC Adv.* **2015,** *5* (127), 104769-104778.

(34) Gao, Y.; Chen, K.; Tan, X.; Wang, X.; Alsaedi, A.; Hayat, T.; Chen, C., Interaction Mechanism of Re(VII) with Zirconium Dioxide Nanoparticles Archored onto Reduced Graphene Oxides. *ACS Sustain. Chem. Eng.* **2017,** *5* (3), 2163-2171.

(35) Xiong, Y.; Cui, X.; Zhang, P.; Wang, Y.; Lou, Z.; Shan, W., Improving Re(VII) Adsorption on Diisobutylamine-Functionalized Graphene Oxide. *ACS Sustain. Chem. Eng.* **2016,** *5* (1), 1010-1018.

(36) Li, J.; Zhu, L.; Xiao, C.; Chen, L.; Chai, Z.; Wang, S., Efficient uptake of perrhenate/pertechnenate from aqueous solutions by the bifunctional anion-exchange resin. *Radiochimica Acta* **2018,** *106* (7), 581-591.

(37) Jia, M.; Cui, H.; Jin, W.; Zhu, L.; Liu, Y.; Chen, J., Adsorption and separation of rhenium(VII) using N-methylimidazolium functionalized strong basic anion exchange resin. *J. Chem. Technol. Biotechnol.* **2013,** *88* (3), 437-443.

(38)Shu, Z.; Yang, M., Adsorption of Rhenium(VII) with Anion Exchange Resin D318. *Chin. J. Chem. Eng.* **2010,** *18* (3), 372-376.

(39) Zhu, L.; Xiao, C.; Dai, X.; Li, J.; Gui, D.; Sheng, D.; Chen, L.; Zhou, R.; Chai, Z.; Albrecht-Schmitt, T. E.; Wang, S., Exceptional Perrhenate/Pertechnetate Uptake and Subsequent Immobilization by a Low-Dimensional Cationic Coordination Polymer: Overcoming the Hofmeister Bias Selectivity. *Environ. Sci. Technol. Lett.* **2017,** *4* (7), 316-322.

(40) Hu, Q. H.; Wang, Y. G.; Gao, X.; Shi, Y. Z.; Lin, S.; Liang, R. P.; Qiu, J. D., Halogen microregulation in metal-organic frameworks for enhanced adsorption performance of ReO_4^-/TcO_4^-. *J. Hazard. Mater.* **2023,** *446*, 130744.

(41) Drout, R. J.; Otake, K.; Howarth, A. J.; Islamoglu, T.; Zhu, L.; Xiao, C.; Wang, S.; Farha, O. K.,

Efficient Capture of Perrhenate and Pertechnetate by a Mesoporous Zr Metal–Organic Framework and Examination of Anion Binding Motifs. *Chem. Mater.* **2018,** *30* (4), 1277-1284.

(42) Li, C. P.; Li, H. R.; Ai, J. Y.; Chen, J.; Du, M., Optimizing Strategy for Enhancing the Stability and $^{99}TcO_4^-$ Sequestration of Poly(ionic liquids)@MOFs Composites. *ACS Cent. Sci.* **2020,** *6* (12), 2354-2361.

(43) Banerjee, D.; Xu, W.; Nie, Z.; Johnson, L. E.; Coghlan, C.; Sushko, M. L.; Kim, D.; Schweiger, M. J.; Kruger, A. A.; Doonan, C. J.; Thallapally, P. K., Zirconium-Based Metal-Organic Framework for Removal of Perrhenate from Water. *Inorg. Chem.* **2016,** *55* (17), 8241-8243.

(44) Banerjee, D.; Elsaidi, S. K.; Aguila, B.; Li, B.; Kim, D.; Schweiger, M. J.; Kruger, A. A.; Doonan, C. J.; Ma, S.; Thallapally, P. K., Removal of Pertechnetate-Related Oxyanions from Solution Using Functionalized Hierarchical Porous Frameworks. *Chem. Eur. J.* **2016,** *22* (49), 17581-17584.

(45) Zu, J.; Ye, M.; Wang, P.; Tang, F.; He, L., Design of a strong-base anion exchanger and its adsorption and elution behavior for rhenium(VII). *RSC Adv.* **2016,** *6* (23), 18868-18873.

(46) Wang, Y.; Xie, M.; Lan, J.; Yuan, L.; Yu, J.; Li, J.; Peng, J.; Chai, Z.; Gibson, J. K.; Zhai, M.; Shi, W., Radiation Controllable Synthesis of Robust Covalent Organic Framework Conjugates for Efficient Dynamic Column Extraction of $^{99}TcO_4^-$. *Chem* **2020,** *6* (10), 2796-2809.

(47) Hao, M.; Chen, Z.; Yang, H.; Waterhouse, G. I. N.; Ma, S.; Wang, X., Pyridinium salt-based covalent organic framework with well-defined nanochannels for efficient and selective capture of aqueous $^{99}TcO_4^-$. *Sci. Bull.* **2022,** *67* (9), 924-932.

(48) Chen, X.-R.; Zhang, C.-R.; Jiang, W.; Liu, X.; Luo, Q.-X.; Zhang, L.; Liang, R.-P.; Qiu, J.-D., 3D Viologen-based covalent organic framework for selective and efficient adsorption of ReO_4^-/TcO_4^-. *Sep. Purif. Technol.* **2023,** *312*, 123409.

(49) Wang, Y.; Lan, J.; Yang, X.; Zhong, S.; Yuan, L.; Li, J.; Peng, J.; Chai, Z.; Gibson, J. K.; Zhai, M.; Shi, W., Superhydrophobic Phosphonium Modified Robust 3D Covalent Organic Framework for Preferential Trapping of Charge Dispersed Oxoanionic Pollutants. *Adv. Funct. Mater.* **2022,** *32* (36), 2205222.

(50) Da, H. J.; Yang, C. X.; Yan, X. P., Cationic Covalent Organic Nanosheets for Rapid and Selective Capture of Perrhenate: An Analogue of Radioactive Pertechnetate from Aqueous Solution. *Environ. Sci. Technol.* **2019,** *53* (9), 5212-5220.

(51) Ding, M.; Chen, L.; Xu, Y.; Chen, B.; Ding, J.; Wu, R.; Huang, C.; He, Y.; Jin, Y.; Xia, C., Efficient capture of Tc/Re(VII, IV) by a viologen-based organic polymer containing tetraaza macrocycles. *Chem. Eng. J.* **2020,** *380*, 122581.
